# Performance of ChatGPT on the Situational Judgement Test—A Professional Dilemmas–Based Examination for Doctors in the United Kingdom

**DOI:** 10.2196/48978

**Published:** 2023-08-07

**Authors:** Robin J Borchert, Charlotte R Hickman, Jack Pepys, Timothy J Sadler

**Affiliations:** 1 Department of Radiology University of Cambridge Cambridge United Kingdom; 2 Department of Radiology Addenbrooke's Hospital Cambridge University Hospitals NHS Foundation Trust Cambridge United Kingdom; 3 Department of General Medicine Lister Hospital East and North Hertfordshire NHS Trust Stevenage United Kingdom; 4 Department of Biomedical Sciences Humanitas University Milan Italy

**Keywords:** ChatGPT, language models, Situational Judgement Test, medical education, artificial intelligence, language model, exam, examination, SJT, judgement, reasoning, communication, chatbot

## Abstract

**Background:**

ChatGPT is a large language model that has performed well on professional examinations in the fields of medicine, law, and business. However, it is unclear how ChatGPT would perform on an examination assessing professionalism and situational judgement for doctors.

**Objective:**

We evaluated the performance of ChatGPT on the Situational Judgement Test (SJT): a national examination taken by all final-year medical students in the United Kingdom. This examination is designed to assess attributes such as communication, teamwork, patient safety, prioritization skills, professionalism, and ethics.

**Methods:**

All questions from the UK Foundation Programme Office’s (UKFPO’s) 2023 SJT practice examination were inputted into ChatGPT. For each question, ChatGPT’s answers and rationales were recorded and assessed on the basis of the official UK Foundation Programme Office scoring template. Questions were categorized into domains of Good Medical Practice on the basis of the domains referenced in the rationales provided in the scoring sheet. Questions without clear domain links were screened by reviewers and assigned one or multiple domains. ChatGPT's overall performance, as well as its performance across the domains of Good Medical Practice, was evaluated.

**Results:**

Overall, ChatGPT performed well, scoring 76% on the SJT but scoring full marks on only a few questions (9%), which may reflect possible flaws in ChatGPT’s situational judgement or inconsistencies in the reasoning across questions (or both) in the examination itself. ChatGPT demonstrated consistent performance across the 4 outlined domains in Good Medical Practice for doctors.

**Conclusions:**

Further research is needed to understand the potential applications of large language models, such as ChatGPT, in medical education for standardizing questions and providing consistent rationales for examinations assessing professionalism and ethics.

## Introduction

ChatGPT is a large language model developed by OpenAI, which uses deep learning to provide responses to natural language input, by identifying the relationships between words and by generating coherent responses [1]. It achieves this in a conversational context following text input and produces an immediate response in an accessible format to users.

These recent advances in language models demonstrate the potentially significant impact of artificial intelligence (AI) technologies on digital health. ChatGPT has already demonstrated its ability to pass professional examinations for postgraduates in the fields of law [2] and business [3]. ChatGPT showed similar promise in the field of medicine [4], and its performance has been assessed on UK-based examinations for medical school admissions [5], as well as those for general practitioners (GPs) [6] and neurologists in training [7].

With regard to the United States Medical Licensing Examination (USMLE), ChatGPT scored at, or near, the pass mark for each step of the examination [4]. Although ChatGPT’s performance has been impressive, the USMLE focuses predominantly on basic science, pharmacology, and pathophysiology (step 1) as well as clinical reasoning and medical management (step 2CK), with less emphasis on other professional skills for becoming a successful doctor [8]. Mbakwe et al [8] argue that ChatGPT’s impressive performance on the USMLE emphasizes the need to develop more relevant approaches to evaluating these crucial skills, which are necessary for doctors but are not assessed in the USMLE. These additional skills are also not assessed in UK-based examinations for which ChatGPT’s performance has already been evaluated, such as the BioMedical Admissions Test [5], the UK Neurology Specialty Certificate Examination [7], and the Applied Knowledge test for GPs [6].

The Situational Judgement Test (SJT) aims to assess many of the skills not covered in the USMLE [4] and in other examinations, which have been assessed using ChatGPT, including communication, teamwork, patient safety, prioritization skills, professionalism, and ethics. At the end of their university studies, all final-year medical students in the United Kingdom applying for Foundation Programme posts (similar to internships in the United States) take the SJT. A candidate’s performance on the SJT accounts for 50% of the overall score for their application to the Foundation Programme, while the other half is calculated from their educational performance in medical school. Later on in their training, many UK doctors are also required to take the Multi-Specialty Recruitment Assessment postgraduate examination that includes a professional dilemmas section similar to those in the SJT. The SJT places emphasis on 4 domains: Knowledge, Skills and Performance; Safety and Quality; Communication, Partnership and Teamwork; and Maintaining Trust - outlined in the General Medical Council’s (GMC) Good Medical Practice [9]. This document lists the essential duties of all doctors working in the United Kingdom. Although performance on the SJT plays a significant role in determining the career path of UK doctors, several reports and student commentaries have suggested that there are significant discrepancies in the correct answers chosen among different experts [10-13].

Our aim was to evaluate the performance of ChatGPT on the SJT and determine how well it performs across the 4 key domains of Good Medical Practice. To our knowledge, this is the first study to investigate the performance of ChatGPT on a situational judgement and professionalism examination of this type.

## Methods

### ChatGPT

The ChatGPT model was trained on vast amounts of data from the internet, up to and including 2021, after which it has not been connected to the internet [14]. Hence, ChatGPT has not been trained on data sets that only became available on the internet from 2022 onward, but it has demonstrated good performance on a range of natural language tasks such as question-answering and text summarization tasks [4,15].

### SJT Examination

The SJT examination is divided into 3 sections, with each question stem first introducing a scenario, followed by a question on how the candidate would approach the situation. These sections include (1) rating the appropriateness or importance of a response, action, or consideration; for example, very appropriate, appropriate, somewhat inappropriate, or inappropriate; (2) multiple-choice questions asking for the 3 most appropriate options from among 8 options; and (3) ranking the appropriateness, or importance, of 5 different actions or considerations in response to a scenario. The SJT is scored on a scale from 0 to 50 points and is not a pass-or-fail examination.

Given the discrepancies in correct answers, and justifications among unofficial study resources, we used the most recent official 2023 SJT practice paper, which is publicly available from the official United Kingdom Foundation Programme Office (UKFPO) website [16], together with a separate document with answers and rationales. This paper would, therefore, not have been available in the training set for ChatGPT as it was released after 2021.

### Encoding

Each question from the 2023 SJT practice paper was formatted identically into the ChatGPT text with the following additions: (1) the official candidate examination instructions were provided before each scenario (Multimedia Appendix 1), and (2) ChatGPT was asked to provide its rationale at the end of each question (Multimedia Appendix 2). A new ChatGPT chat session was started for each question and, therefore, the instructions were written in the singular form to reflect that the model was being asked to answer each question separately to reduce the risk of memory retention bias.

### Assessing Performance

We used the official UKFPO scoring templates to determine the number of marks scored by ChatGPT in each of the 3 sections of the examination. The scoring for each question is not binary, and partial marks are awarded for answers that are nearly correct. For example, in the multiple-choice section, each question has 3 correct answers from a choice of 8 options; each correct answer is awarded 4 points with a maximum of 12 points per question. Therefore, a candidate can score 0, 4, 8, or 12 marks for each multiple-choice question. The rating and ranking sections award partial marks for an answer that is close to the correct one. ChatGPT’s performance was calculated as a percentage for each section using the official UKFPO scoring templates. We also determined the proportion of questions for which the answers were correct (defined as scoring 100% of the available marks for the given question), mostly correct (50%-99%), and mostly incorrect (<50%) for each section.

The final SJT score provided to candidates is on a scale from 0 to 50, which is based on test-equating and scaling the raw marks achieved on the paper. This conversion formula varies between sittings and is not made publicly available by the UKFPO. We, therefore, reported ChatGPT’s performance as a percentage instead of reporting it on the 0-50–point scale, which is normally used to compare performance between human candidates. Both the SJT and Educational Performance Measure scores determine a final-year medical student’s ranking when applying to the Foundation Programme. The Educational Performance Measure is a measure of performance in medical school up to the point of application to the Foundation Programme with students grouped into deciles.

### Good Medical Practice Guidelines

In order to assess ChatGPT’s performance across the different domains of Good Medical Practice, each question was categorized into at least 1 domain. To classify the questions, we used the 2023 practice paper answer sheet provided by the UKFPO, which also contains the rationale for most answers. Many of the rationales contained direct references to at least 1 domain from the Good Medical Practice guidelines that were used for categorization. Questions with rationales, which had missing links to the domains, were categorized by 2 independent reviewers on the basis of both the question itself and the rationale provided by the UKFPO. Both reviewers recently completed the Foundation Programme on which this SJT examination is based. The reviewers were blinded to each other’s categorization of each question, and disagreements were resolved by a third reviewer who was a consultant radiologist within the National Health Service and was blinded to the categorizations made by the 2 initial reviewers. Once all questions in the examination were assigned domains combining the rationales offered by the UKFPO and the screening approach used for the remaining questions, ChatGPT’s performance in each domain of Good Medical Practice was assessed using the official scoring templates and reported as a percentage.

A summary of the workflow for this study including sourcing, encoding, adjudicating results, and assessing performance can be found in Table 1.

**Table 1:** A schematic workflow of sourcing, encoding, adjudicating results, and assessing performance for this study.

Workflow step	Description
Sourcing	Official 2023 UKFPO^a^ SJT^b^ practice paper with questions, answers and rationales for each answer
Encoding in ChatGPT	The following was inputted into ChatGPT for each question: Official candidate examination instructions Question from the practice paper “Provide your rational for each answer”
Adjudicating results	Official UKFPO scoring templates used as a reference for correct answers
Assessing performance	Percentage of total possible marks Proportion of questions for which the answers were correct (100%), mostly correct (50%-99%), and mostly incorrect (<50%) Percentage of total possible marks within each domain of Good Medical Practice

^a^UKPFO: UK Foundation Programme Office.

^b^SJT: Situational Judgement Test.

### Ethical Considerations

This study did not involve human or animal participants and ethics approval was not required.

## Results

### Overall Performance

Overall, ChatGPT scored 76% (929 of a possible 1217 marks) on this SJT examination (Multimedia Appendix 3).

**Figure 1:**
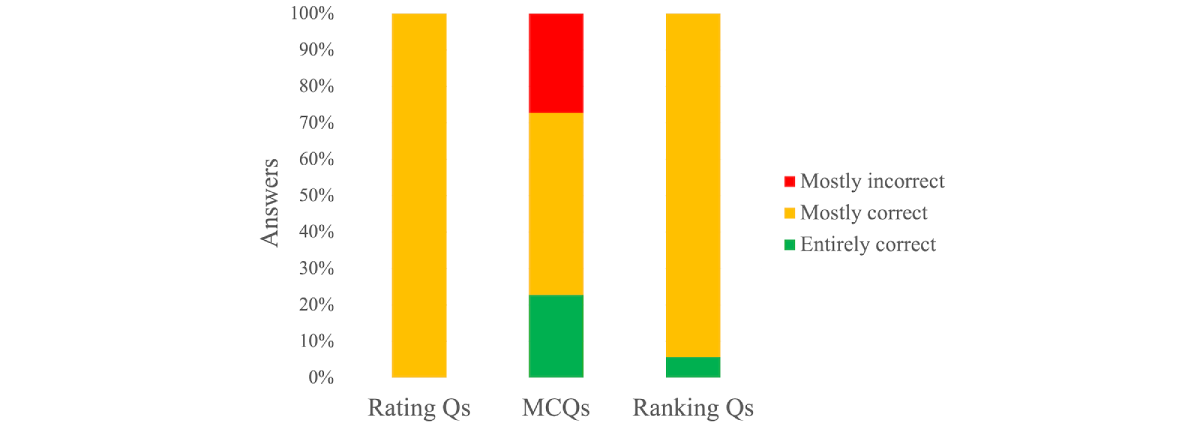
ChatGPT’s performance in each section of the examination depicting the proportion of entirely correct (100%), mostly correct (50%-99%), or mostly incorrect answers (<50%). MCQ: multiple-choice question. Q: question.

For the rating section of the examination, ChatGPT scored 78% (197/253 marks) with 0% (0/18 questions) entirely correct, 100% (18/18 questions) mostly correct, and 0% (0/18 questions) mostly incorrect responses (Figure 1).

For the multiple-choice section of the examination, ChatGPT scored 65% (172/264 marks) with 23% (5/22 questions) entirely correct, 50% (11/22 questions) mostly correct, and 27% (6/22 questions) mostly incorrect responses (Figure 1).

For the ranking section of the examination, ChatGPT scored 80% (560/700 marks) with 6% (2/35 questions) entirely correct, 94% (33/35 questions) mostly correct, and 0% (0/35 questions) mostly incorrect responses (Figure 1).

### Good Medical Practice Domains

There were 170 questions and answer statements that were classified into at least one of the GMC domains. Of these, 84 (49% of the total) were explicitly linked to a GMC domain within the rationale provided in the UKFPO’s official answer sheet. The independent reviewers then screened the remaining 86 and agreed on which GMC domains they applied to for 76 (88%) of them. The remaining 10 were then assessed by the tiebreaker (consultant radiologist), and their labels were used for the analysis.

ChatGPT scored 78% (328/419) in the Knowledge, Skills and Performance domain, 76% (484/635) in the Safety and Quality domain, 76% (340/448) in the Maintaining Trust domain, and 75% (784/1046) in the Communication, Partnership and Teamwork domain.

### Answers With the Biggest Discrepancies

In the rating section, ChatGPT’s worst performance was noted on a question related to the appropriateness of specific actions after discovering that a medical student has likely acquired detailed information about the scenarios that will feature in an upcoming examination. The official answers and rationale advise that it would be (1) somewhat appropriate to inform the medical student that their Educational Supervisor will be informed about the situation and (2) very appropriate to encourage the student to inform the medical school that they have acquired information about the examination. ChatGPT labeled these options as inappropriate and somewhat inappropriate, respectively, with the rationale that (1) “Threatening to inform her Educational Supervisor about her behaviour is not a productive or supportive approach…It is important to remember that as a facilitator, the doctor’s role is to support and guide the student in their learning, not to police their behaviour” and (2) “While it is important [for the student] to be honest about how she obtained information, encouraging her to declare this to the medical school may be premature at this point. It may be more appropriate to first have a conversation with [the student] to understand why she sought additional information and to provide guidance on appropriate conduct.”

In the ranking section of the examination, ChatGPT scored its lowest marks (50%) on a question asking about the appropriateness of certain actions when one has arrived home after one’s shift and realizes that they forgot to handover an urgent blood sample that needs to be taken today. ChatGPT deemed returning to the ward immediately to perform the blood test as the most appropriate action, whereas the official marking labeled this is as one of the less appropriate options. ChatGPT also ranked telephoning the ward and leaving a message with the nursing team as a less appropriate option because “the nursing team may not have the necessary information or authority to take appropriate action for the patient,” while the official marking classified this as one of the more appropriate actions.

## Discussion

### Principal Findings

We evaluated the performance of ChatGPT on the SJT: a national examination for final-year medical students in the United Kingdom, which assesses attributes including communication, teamwork, patient safety, prioritization skills, professionalism, and ethics. Overall, ChatGPT scored 76% on the examination. It answered 0%, 23%, and 6% of the questions entirely correctly in the rating, multiple-choice, and ranking sections of the examination, respectively, but was mostly correct for 100%, 50%, and 94% of the questions in these sections. ChatGPT scored consistently across the 4 key domains of Good Medical Practice.

ChatGPT’s overall performance was impressive considering that it was correct or mostly correct for the majority of questions in the examination. However, the proportion of the questions that were answered with 100% accuracy was lower than expected with its best performance being in the multiple-choice section, in which it chose the 3 correct options in approximately one-fourth of the questions. This could be due to flaws in ChatGPT’s reasoning in some of these situations. However, ChatGPT’s low proportion of entirely correct (100%) answers may also reflect inconsistencies within the examination itself. Several reports and student commentaries have suggested that there are significant discrepancies in the correct answers chosen by different experts [10-13]. If this is the case, the inconsistencies in the rationale underlying different questions and the official answers offered by the UKFPO may contribute to worse performance by ChatGPT on the examination. It is interesting to note that for some of the answers where ChatGPT significantly deviated from the official UKFPO answers, ChatGPT’s rationale for its answers came across as reasonable and insightful and would likely resonate with many candidates compared to the official answers provided by the UKFPO. It also raises the question of how large language models, such as ChatGPT, could be used to help standardize these types of situational judgement and professionalism examinations, by providing consistent answers and rationale throughout. In this context, ChatGPT could also serve as a preparation tool for prospective SJT candidates, although it is important to consider whether the ethical implications of this technology could widen disparities. For example, concerns have been raised regarding differential attainment between candidates from different ethnic groups with SJT questions potentially enforcing cultural biases [13]. ChatGPT and other AI language models may inherit biases from the data that they are trained on [17] and, hence, may reinforce these cultural biases in the context of the SJT. Access to these technologies, both in terms of awareness and financial capacity may also further widen these disparities in performance instead of promoting equality and ensuring that the test is solely assessing aptitude.

Interestingly, ChatGPT scored 65% in the multiple-choice section versus 78% and 80% in the rating and ranking sections, respectively. This may reflect that this large language model is better suited to tasks that involve ranking and prioritization rather than selecting from a list of most appropriate, or relevant, options for a given scenario. ChatGPT has been trained on a wide gamut of data available from the internet, which may not always be factually correct, but amalgamated together means that the model may be more competent at dealing with open-ended questions which involve listing options in order of importance or relevance, as opposed to questions with individual correct answers.

ChatGPT performed consistently across the 4 domains of Good Medical Practice, having scored between 75% and 78% across them. ChatGPT performed slightly better in the Knowledge, Skills and Performance and Safety and Quality domains than in the Communication, Partnership and Teamwork domain. We speculate that this could be explained by questions pertaining to knowledge and safety being more objective in nature, whereby patient safety and delivering high-quality care are always prioritized. These types of scenarios may provide ChatGPT with a more straightforward approach to classifying the appropriateness of the options, compared to questions pertaining to communication and teamwork where decision-making is more subjective and nuanced. However, the differences in ChatGPT’s performance across these domains were too small to provide more definitive insight.

### Limitations

There were several limitations in this study: First, in practice, the raw score for this examination is converted to a 0-50–point scale, which is based on test-equating and a scaling conversion method that is not publicly available. We also do not have access to the results of medical students taking this practice examination and are therefore unable to directly compare ChatGPT’s performance to that of final-year medical students. Second, the answer sheet and rationales provided by the UKFPO for this examination only explicitly linked 49% of the questions and answer statements to the GMC domains outlined in Good Medical Practice. We therefore devised a method to link the remaining questions to the domains, which involved 2 independent reviewers and a tiebreaker, the results of which may have differed from those of the UKFPO. Third, many questions pertained to more than 1 domain of Good Medical Practice; hence, there was an overlap in questions across different domains when assessing ChatGPT’s performance in each domain. Fourth, our search was run on the February 2023 version of ChatGPT, and given the constant development of this large language model, future iterations may yield different outcomes.

### Conclusions

Overall, ChatGPT performed well in the examination but scored 100% for only a few questions, which may reflect inconsistencies in the examination or errors in ChatGPT’s reasoning (or both). This builds on the existing literature by demonstrating that AI-driven large language models such as ChatGPT not only perform well on a wide range of clinically based examinations, but also offer, for the most part, rational responses to professional and ethical dilemmas faced by doctors. Future research should focus on identifying patterns and inconsistencies in the ethical approaches of AI language models and mitigating potential biases in them. Directly comparing the performance of these types of models with that of human candidates in relation to situational judgement dilemmas will provide more direct insight into their performance relative to that of humans. If the ethical foundations of models such as ChatGPT are deemed appropriate and reliable, it would provide the opportunity for integration directly into medical education with, for example, interactive platforms, simulated scenarios related to situational judgement, and personalized feedback, as well as standardization of examinations. Finally, in order to achieve this, it will be crucial to use a collaborative approach among experts in AI, medicine, and medical education to realize the full potential of these new technologies. Addressing these points will help develop this field and promote the integration of large language models, such as ChatGPT, into medical education, thus helping to standardize assessments that evaluate professionalism and ethics while maintaining high-quality and equitable medical education standards.

## Appendix

Multimedia Appendix 1 Situational Judgement Test templates.

Multimedia Appendix 2 Supporting information—ChatGPT output.

Multimedia Appendix 3 Raw scores and GMC domains for each question in the SJT exam. GMC: General Medical Council; SJT: Situational Judgement Test.

## Abbreviations

AI: artificial intelligence
GMC: General Medical Council
GP: general practitioner
SJT: Situational Judgement Test
UKFPO: UK Foundation Programme Office
USMLE: United States Medical Licensing Examination
