# Supplementary material for: Performance of ChatGPT on the Situational Judgement Test—A Professional Dilemmas–Based Examination for Doctors in the United Kingdom
Source: JMIR Med Educ. 2023 Aug 7;9:e48978. doi: 10.2196/48978 (PMC10442724; doi:10.2196/48978)
Supplement: Multimedia Appendix 1 [file mededu_v9i1e48978_app1.docx]

**Rating template:**

You will be presented with a typical scenario that Foundation Year One (FY1) Doctors encounter, and you will be asked a question about dealing with the scenario. When answering the questions, please consider yourself to be a FY1 Doctor and please answer based on what you should do. You may feel you would like more information before answering, but please answer the question based only on the information provided.

[Scenario and question copied directly from UKFPO 2023 SJT Practice Paper]

[Answer options]

Provide your rationale for each answer.

**Multiple choice template:**

You will be presented with a typical scenario that Foundation Year One (FY1) Doctors encounter, and you will be asked a question about dealing with the scenario. When answering the questions, please consider yourself to be a FY1 Doctor and please answer based on what you should do. You may feel you would like more information before answering, but please answer the question based only on the information provided.

[Scenario and instructions copied directly from UKFPO 2023 SJT Practice Paper]

[Answer options A-H]

Provide your rationale for each answer.

**Ranking template:**

You will be presented with a typical scenario that Foundation Year One (FY1) Doctors encounter, and you will be asked a question about dealing with the scenario. When answering the questions, please consider yourself to be a FY1 Doctor and please answer based on what you should do. You may feel you would like more information before answering, but please answer the question based only on the information provided.

[Scenario and question copied directly from UKFPO 2023 SJT Practice Paper]

[Answer options A-E]

Provide your rationale for each answer.
