# Supplementary material for: Performance of ChatGPT on the Situational Judgement Test—A Professional Dilemmas–Based Examination for Doctors in the United Kingdom
Source: JMIR Med Educ. 2023 Aug 7;9:e48978. doi: 10.2196/48978 (PMC10442724; doi:10.2196/48978)
Supplement: Multimedia Appendix 2 [file mededu_v9i1e48978_app2.docx]

Supporting information – ChatGPT output

**Answers and rationale for rating questions:**

1

A. "Please be assured that I will try my best to find out the cause of your pain." - 1 (Very appropriate) This response acknowledges the patient's concern and reassures them that the doctor will do their best to find the cause of their pain. It also shows empathy towards the patient's previous experiences and builds trust.

B. "I’m here to help you, please don’t speak to me in that way." - 4 (Inappropriate) This response is defensive and confrontational. It does not address the patient's concerns and may escalate the situation. It is important for doctors to remain professional and not take such comments personally.

C. "Let me see if a senior doctor is available to assess you instead." - 1 (Very appropriate) This response shows respect for the patient's wishes and acknowledges their concern. It also allows for the patient to be seen by a more experienced doctor, which may help to address their concerns and build trust.

D. "I’m sorry you feel that way, but it is not my fault you are still experiencing pain." - 4 (Inappropriate) This response is defensive and dismissive. It does not acknowledge the patient's concerns and may further undermine their confidence in the doctor's ability to treat them.

E. "I can see if a senior doctor is available, however, you might end up having to wait a while to be seen." - 2 (Somewhat appropriate) This response acknowledges the patient's request and offers a possible solution, but it also highlights the potential negative consequence of waiting for a more experienced doctor. It is important for doctors to be honest with patients, but this response may increase anxiety and worry for the patient.

2

A. Provide Sarah with some publicly available information about medical school examination - 1 (Very appropriate)

It is perfectly reasonable to provide publicly available information about the medical school examination to Sarah, as this will help her prepare for the exam. As a facilitator, it is also part of your duty to help students prepare for the exam.

B. Explain to Sarah that it would be unfair on other candidates if only she knew the scenarios before the clinical exam - 3 (Inappropriate)

This response may come across as accusatory or condescending to Sarah and may not provide her with any helpful information. Moreover, it is not relevant to the question that she asked.

C. Reassure Sarah that she should not need to know the scenarios before the clinical exam in order to do well - 1 (Very appropriate)

This is an appropriate response as it helps to reassure Sarah and may alleviate any anxiety she has about the exam. Additionally, it is a true statement as the clinical exam is designed to test a student's ability to think critically and apply their knowledge to a range of clinical scenarios, not just those that they may have been explicitly told about beforehand.

In conclusion, options A and C are the most appropriate responses. Option B is not relevant to the question asked and may come across as accusatory or condescending.

3

A. Ask Sarah why she has chosen to also ask James for information - 2 (Somewhat inappropriate)

It may be reasonable to ask Sarah why she sought information from James as well, but it could come across as confrontational or accusatory. It is possible that Sarah simply wanted a second opinion or clarification on certain points. Therefore, it may be better to approach this conversation with an open mind and seek to understand why Sarah sought additional information.

B. Tell Sarah that you will need to inform her educational supervisor* about the situation - 4 (Very inappropriate)

Threatening to inform Sarah's educational supervisor about her behavior is not a productive or supportive approach. It may lead to mistrust and damage the relationship between the doctor and the student, which could make it difficult for the doctor to work effectively with the student in the future. It is important to remember that as a facilitator, the doctor's role is to support and guide the student in their learning, not to police their behavior.

C. Encourage Sarah to declare to the medical school that she has been told information about the clinical exam - 3 (Somewhat inappropriate)

While it is important for Sarah to be honest about how she obtained information, encouraging her to declare this to the medical school may be premature at this point. It may be more appropriate to first have a conversation with Sarah to understand why she sought additional information and to provide guidance on appropriate conduct. If there is a concern about academic misconduct or unethical behavior, this may need to be escalated to the appropriate authorities.

D. Explore with Sarah why she feels she needs to know the information - 1 (Very appropriate)

This approach is a good way to start a conversation with Sarah about her behavior. It demonstrates an interest in understanding her perspective and can provide an opportunity to offer guidance and support. The doctor can ask questions to explore Sarah's motivations for seeking information from James and provide feedback on appropriate behavior in this situation. This approach is supportive and non-judgmental, which can help maintain a positive relationship with the student.

4

A. "It is up to you whether or not you go home." - 2 (Somewhat appropriate) Rationale: While it is important to respect the patient's autonomy and their decision about their own care, this statement on its own could be seen as dismissive or uncaring. It may also give the impression that the doctor does not want to engage further with the patient to explore their concerns.

B. "Is there a particular reason why you do not want to have surgery?" - 1 (Very appropriate) Rationale: This is a very appropriate response as it demonstrates a willingness to listen to the patient and explore their concerns. It is important to understand the patient's perspective and to address any fears or misconceptions they may have about the surgery.

C. "Do you have any family members or friends who can come to support you?" - 1 (Very appropriate) Rationale: This response shows concern for the patient's emotional well-being and provides the opportunity for the patient to receive support during this difficult time. Family members or friends can be a valuable source of support and can help the patient make informed decisions about their care.

5

A. “You know that this is confidential information that I cannot discuss with you.” - 1 This response is appropriate as it clearly explains to the nurse that patient information is confidential and cannot be shared with anyone who is not the legal guardian. It also shows professionalism and adherence to patient confidentiality laws.

B. “You will need to speak with your niece’s parents directly about this.” - 1 This response is also appropriate as it directs the nurse to the appropriate person who can provide information about the patient. It shows professionalism and adherence to patient confidentiality laws.

C. “Try not to worry. I’m sure everything is going to be fine.” - 3 This response is not appropriate as it does not address the nurse’s concerns or the question asked. It is also not professional as it provides no information about the patient's condition or treatment.

D. “It is inappropriate for you to ask this of me so please do not ask me again.” - 4 This response is inappropriate as it is confrontational and may damage the working relationship between the nurse and the doctor. It does not address the nurse's concerns or the question asked, and it does not provide any guidance on how to obtain the necessary information.

6

A. Ask the nurse in charge for advice about how to respond - 1 (Very appropriate) It is important to seek advice from a more senior member of staff in situations where there may be a breach of patient confidentiality. The nurse in charge may be able to provide guidance on how to handle the situation without compromising patient confidentiality or damaging the relationship with the nurse in question.

B. Tell the patient’s parents that you suspect the nurse was reading their daughter’s notes - 4 (Inappropriate) It would be inappropriate to discuss suspicions of a breach of patient confidentiality with the parents without first gathering more information and seeking advice from a more senior member of staff. Additionally, it is important to avoid causing undue distress to the parents.

C. Ask the nurse why he was reading a set of patient notes for a ward he does not work on - 2 (Somewhat inappropriate) While it may be appropriate to question the nurse about his actions, it is important to do so in a sensitive and non-confrontational manner. It may be better to seek advice from a more senior member of staff before approaching the nurse in question.

7

A. The impact on the rest of the ward staff if you leave work now - 2 (Somewhat appropriate) While it is important to consider the impact of leaving work early on the rest of the ward staff, this response may not be the most effective in this situation. The priority should be patient safety and the well-being of the doctor. It may be more appropriate to focus on those factors in the conversation with the clinical supervisor.

B. The risk to patient safety if you complete your shift feeling like you do - 1 (Very appropriate) This response is very appropriate as patient safety should be the top priority in any medical setting. It is important to communicate any concerns about being able to provide safe care to patients. The clinical supervisor may be more likely to respond positively if they understand the potential risk to patient safety.

C. The amount of time you have left until the end of your shift - 4 (Inappropriate) This response may not be helpful in the situation as it does not address the concerns that the doctor is experiencing. The amount of time left in the shift is less important than the doctor's ability to provide safe care to patients.

8

A. Reassure the parents that the delay in giving the drug will not cause harm to their child - 1 (Very appropriate) It is appropriate to reassure the parents that delaying the drug for half an hour will not harm the child. As the timing of the dose is not critical, it is important to ease the parents' concerns and explain that the child is not in danger.

B. Tell the parents that the delay is because the nurse thinks that your drug calculations are incorrect - 4 (Inappropriate) It would not be appropriate to involve the parents in a disagreement between healthcare professionals. This would likely cause unnecessary stress and anxiety for the parents and may damage their trust in the healthcare team.

C. Ask the consultant to confirm with yourself and the nurse what dose to give to the patient - 1 (Very appropriate) As the nurse has expressed concerns about the drug dose, it is appropriate to seek further clarification from the consultant. Asking the consultant to confirm the dose with both yourself and the nurse will help to ensure that the correct dose is administered to the patient.

D. Avoid having a conversation with the parents until after you have sorted out the issue with the drug dose - 3 (Somewhat inappropriate) While it is important to resolve the issue with the drug dose, it is not appropriate to avoid speaking to the parents altogether. Communication is essential in building trust with patients and their families, and it is important to keep the parents informed about their child's care. It would be better to explain that there is a delay in giving the drug and that you are working to resolve the issue.

9

A. “The consultant asked for the chest x-ray but I’m not sure why.” Rating: 2 (Somewhat inappropriate)

Rationale: While it is honest, this response does not provide the patient with any useful information and may leave them feeling confused and uncertain about their care. As a healthcare professional, it is important to communicate with patients in a clear and compassionate way, even if you are uncertain about the reason for a particular test or treatment.

B. “I’m not sure why you need one if you had one yesterday; I’ll cancel the chest x-ray.” Rating: 4 (Inappropriate)

Rationale: It is not appropriate to cancel a test that has been ordered by a consultant without discussing it with them first. Additionally, the patient may need the chest x-ray for a new or worsening condition that has developed since their previous one. As a healthcare professional, it is important to follow orders from consultants and communicate with them if there are concerns or questions about a patient's care.

C. “I’ll check that the consultant realizes that you had a chest x-ray yesterday.” Rating: 1 (Very appropriate)

Rationale: This response is the most appropriate as it shows concern for the patient and a willingness to follow up on their concerns while still respecting the consultant's orders. It is important to communicate with the consultant and ensure that the patient's care is being coordinated effectively. By doing so, the patient can feel reassured that their concerns have been heard and addressed.

10

A. That Helen and her partner are having a private conversation - 4 (Inappropriate) It is important to respect patients' and their families' privacy, but in this case, it is necessary to intervene because the partner's behavior is becoming aggressive and may pose a risk to Helen's safety. Ignoring the situation could potentially escalate the situation and compromise Helen's care.

B. That Helen’s patient confidentiality is at risk of being compromised - 3 (Somewhat inappropriate) While it is important to protect patient confidentiality, this response may not be the most appropriate in this situation as it does not address the issue at hand. It is important to prioritize the safety of the patient and intervene if the situation is escalating.

C. That Helen’s partner may become physically aggressive - 1 (Very appropriate) This response is highly appropriate as it acknowledges the potential risk to the patient's safety and prompts immediate action to ensure that the situation does not escalate. This response should be followed up with a call for assistance from other members of the healthcare team.

D. That it is not your job to intervene between patients and their families - 4 (Inappropriate) While it is important to respect patients' and their families' autonomy, it is also important to ensure that the patient's safety and well-being are not compromised. In this situation, the partner's behavior is escalating, and intervention is necessary to ensure that the situation does not become violent.

11

A. “Was it necessary to change the rota?” - 3 (Inappropriate) This response may come across as confrontational or accusatory towards Dr Wong. It could create a negative atmosphere and damage the working relationship with the consultant. It would be better to approach the conversation with an open mind and a willingness to listen to Dr Wong's reasoning for the change.

B. “It might be a good idea to discuss the new rota at a meeting with the whole team.” - 1 (Very appropriate) This response is appropriate as it suggests a solution that involves the entire team. By discussing the rota at a meeting, everyone can share their thoughts and concerns, and a collaborative solution can be found. It also shows initiative in trying to improve team morale and communication.

C. “I think it's OK, but you should speak to the rest of the team to see what they think." - 2 (Somewhat appropriate) This response is somewhat appropriate as it acknowledges that there may be differing opinions on the rota change. It suggests that Dr Wong should seek out the opinions of others, which is good advice. However, it could also be seen as passing the responsibility onto Dr Wong and not taking an active role in addressing the issue. It would be better to offer to help facilitate a meeting or discussion among the team.

12

A. Ignore the nurses, leaving the staff room to eat your lunch elsewhere - Inappropriate (4) This response would not be appropriate as it ignores the potential issue with Eliza's behaviour and does not address the negative impact it is having on others in the workplace.

B. Find your FY2 colleague to tell her what you heard the nurses saying about her - Inappropriate (4) This response would not be appropriate as it could further exacerbate the situation and cause unnecessary conflict between Eliza and the nurses. It may also make Eliza feel more isolated and unsupported.

C. Speak to your FY2 colleague to find out if there is a reason for the recent change in her behaviour - Very appropriate (1) This response would be appropriate as it shows concern for Eliza's wellbeing and seeks to understand the reason behind her change in behaviour. It is important to approach Eliza with an open mind and in a non-judgemental way, to show that you are there to support her.

D. Advise your FY2 colleague that you have observed a change in behaviour that is having a negative impact at work - Very appropriate (1) This response would be appropriate as it directly addresses the issue and the impact that Eliza's behaviour is having on others in the workplace. It also allows Eliza to understand the potential impact of her behaviour and work towards addressing it. However, it is important to approach this conversation in a sensitive and supportive manner.

13

A. Reassure your FY2 colleague that she will feel less stressed when the exam is over - 2 (Somewhat inappropriate) While it is important to be empathetic and offer support to colleagues, simply reassuring someone that their stress will go away after an event can be dismissive of their current experience. This response may also come across as trivializing the stress of the exam and not taking Eliza's concerns seriously.

B. Ask your FY2 colleague if she has considered speaking to her educational supervisor about how she is feeling - 1 (Very appropriate) It is appropriate to suggest that Eliza speaks to her educational supervisor as this individual is responsible for her training and well-being. Speaking to the educational supervisor can provide additional support and resources that may not be available to colleagues. It is important to encourage Eliza to seek the help that is available to her and support her in doing so.

C. Suggest taking on some of your FY2 colleague’s workload to allow her more time to study - 3 (Somewhat inappropriate) While it is important to be supportive, taking on someone else's workload can result in additional stress and burnout. This response may also inadvertently send the message that Eliza is not capable of handling her workload and may harm her professional development. It is important to suggest more appropriate ways to manage her workload or seek additional support.

D. Inform your FY2 colleague that some of the nurses have noticed a change in her behavior - 3 (Somewhat inappropriate) While it is important to address behavioral changes in colleagues, informing Eliza that some of the nurses have noticed a change in her behavior may not be the most appropriate approach. This response may come across as accusatory or confrontational, and may not encourage Eliza to seek help or support. It is important to approach the situation with sensitivity and empathy, and focus on offering support and resources to help Eliza manage her stress and workload.

14

A. That the ward nurse in charge must have a reason for wanting you to stay - 1 (Very appropriate): This response is appropriate because it acknowledges the nurse's concerns and shows a willingness to understand the situation. It also opens up the opportunity for the nurse to provide any information or concerns that they may have.

B. That the consultant probably knows the requirements of both wards the best - 1 (Very appropriate): This response is also appropriate because it shows a willingness to defer to the consultant's judgement, who has a broader view of the situation and can make decisions that are in the best interest of both wards.

C. That you might have an easier afternoon if you stay on your current ward - 3 (Inappropriate): This response is inappropriate because it prioritizes personal convenience over the needs of patients and the healthcare team. It also shows a lack of willingness to assist a team in need.

D. That your relationship with the ward nurse in charge might be negatively affected if you go to the other ward - 4 (Inappropriate): This response is inappropriate because it puts personal relationships above patient care and the needs of the healthcare team. It can also be seen as manipulative, implying that the nurse's approval should be a higher priority than the needs of the patients.

15

A. “Don’t worry, your father will be transferred next week.” - Inappropriate (4) This response is inappropriate because it is not certain that the patient will be transferred next week. The consultant has explained that it is not safe for the patient to travel for at least another week. The response could raise the patient and family's expectations and lead to disappointment if the transfer does not happen as planned.

B. “Your father’s health is the most important thing to consider in deciding when he can be transferred.” - Very appropriate (1) This response is very appropriate because it acknowledges the daughter's concerns but also highlights the importance of the patient's health. It is important to prioritize the patient's safety and well-being over the convenience of the family. This response also reassures the daughter that the patient's care is being taken seriously.

C. “It is not necessary for you to visit every day, I am sure he would be OK.” - Inappropriate (4) This response is inappropriate because it dismisses the daughter's concerns and emotions. The daughter may feel guilty or upset if she cannot visit her father every day, and this response could make her feel unsupported. Additionally, the response does not address the issue of transferring the patient closer to home.

D. “Could you speak with your father on the phone, rather than visiting so often?” - Appropriate (3) This response is appropriate because it offers a potential solution to the daughter's concerns. If the daughter is feeling exhausted from traveling to the hospital every day, speaking with her father on the phone could be a way to stay in contact without the physical strain of travel. However, this response does not address the issue of transferring the patient closer to home, which may still need to be considered.

16

A. Apologise to Mrs McDermott for causing confusion following Jeremy's comment - This response is appropriate (1). Apologizing to the patient for any confusion caused by Jeremy's comment is important as it shows empathy towards the patient and also acknowledges that the patient's understanding is a priority. It also helps to reassure the patient that their care is being handled competently.

B. Ignore Jeremy’s comment and continue the conversation with Mrs McDermott - This response is inappropriate (4). Ignoring Jeremy's comment and continuing the conversation may give the impression that the patient's concerns or questions are not important. It could also make the patient feel uncomfortable or less valued.

C. After the consultation, explain to Jeremy that it was an inappropriate time to bring up the article that he has read online - This response is appropriate (2). Explaining to Jeremy after the consultation that it was not an appropriate time to bring up the article he read online helps to guide him on professional conduct and ethical behavior. However, it is important to note that feedback should be given in a constructive manner and at an appropriate time.

D. Ask Jeremy to explain to the patient why he believes that the alternative drug he has suggested is more effective - This response is inappropriate (3). As a medical student, Jeremy may not have the authority to recommend treatment to patients directly. Additionally, it could confuse the patient and affect their trust in the medical team, especially when there is a change of plan. It is better to explain to Jeremy after the consultation why it is not appropriate for him to recommend alternative drugs to patients.

17

A. Explain to Raphael that you feel overwhelmed, under pressure, and need his support - 1 (Very appropriate) Rationale: It is appropriate to communicate the situation with Raphael and ask for his support. As colleagues, it is important to work together to ensure safe patient care. By expressing your concern, Raphael may understand the seriousness of the situation and provide assistance.

B. Explain to Raphael that his behavior is inappropriate - 3 (Inappropriate) Rationale: While it may be frustrating and tempting to express disappointment or anger towards Raphael's behavior, it is not appropriate to label his behavior as inappropriate. It is important to address the situation constructively, rather than putting Raphael on the defensive.

C. Leave Raphael to sleep and carry on alone throughout the shift - 4 (Inappropriate) Rationale: Leaving Raphael to sleep while you struggle alone is not a safe or appropriate solution. Patient care may be compromised, and it could lead to burnout or errors on your part. It is important to address the situation and find a solution that ensures safe and effective patient care.

D. Ask other colleagues on the ward for their support - 1 (Very appropriate) Rationale: Asking other colleagues for their support is a good way to distribute the workload and ensure safe patient care. The team should work together to ensure that all tasks are completed and patients are well cared for.

E. Inform a more senior colleague that you are not receiving the support that you need from your FY2 - 1 (Very appropriate) Rationale: If Raphael is not providing the support needed to ensure safe patient care, it is appropriate to escalate the situation to a more senior colleague. They may be able to provide additional support or reassign tasks to ensure patient safety.

F. Decide to work late to finish the tasks - 4 (Inappropriate) Rationale: Working late to finish the tasks is not an appropriate solution as it could lead to errors, burnout, or fatigue, which may affect patient care. It is important to address the situation and find a solution that ensures safe and effective patient care.

18

A. Ask Raphael why he is struggling to sleep during his time off - 1 (Very appropriate) It is important to understand the underlying reason for Raphael's sleep deprivation. Asking him why he is struggling to sleep during his time off may help identify if there is an underlying medical condition or if there are external factors contributing to his sleeplessness. This information can help in finding an appropriate solution for his problem.

B. Tell Raphael that he is putting patients at risk by coming to work over-tired - 2 (Somewhat appropriate) While it is important to emphasize the potential risks associated with working while sleep-deprived, it may not be the most effective approach. This statement may come off as accusatory and may not encourage Raphael to seek help or change his behavior.

C. Encourage Raphael to seek support from his General Practitioner (GP)* - 1 (Very appropriate) Encouraging Raphael to seek support from his GP is an appropriate response. His GP can help identify underlying medical conditions or suggest lifestyle changes that may help with his sleep deprivation.

D. Advise Raphael that you think it is inappropriate to sleep whilst on shift - 3 (Somewhat inappropriate) While it is generally inappropriate for healthcare professionals to sleep on shift, this may not be the most effective response to the situation at hand. Raphael has already slept on shift, and this statement may come off as judgmental and may not lead to any positive changes or help for Raphael. A more constructive approach would be to encourage Raphael to seek help for his sleep deprivation.

**Answers and rationale for multiple choice questions:**

19

The most appropriate actions to take in this situation are B, C, and G.

Option B: Inform your consultant that the warfarin prescription is against the hospital protocol. This option is important as it is essential to maintain patient safety by following hospital protocols and guidelines. As a doctor, it is our responsibility to ensure that the treatment plan is in the patient's best interest, and following the hospital protocol is an integral part of it. By informing the consultant, we can address the issue and ensure that the patient's safety is not compromised.

Option C: Ask your consultant if she wants you to change the prescription to a smaller dose. This option is also crucial as it involves communicating with the consultant and seeking clarification on the prescription. By doing so, we can ensure that the consultant is aware of the hospital protocol and can make an informed decision on whether to change the prescription or not.

Option G: Ask a different senior colleague for advice on how to proceed with the prescription. This option is essential as it involves seeking advice from a more senior colleague who may have more experience in dealing with such situations. By doing so, we can ensure that we make an informed decision that is in the patient's best interest.

The other options are not appropriate in this situation.

Option A: Asking the pharmacist to supply the high dose of warfarin, as per the prescription, goes against the hospital protocol and puts the patient's safety at risk.

Option D: Asking the patient whether he is willing to accept the risks associated with the higher dose is not appropriate as it is the doctor's responsibility to ensure that the treatment plan is safe for the patient.

Option E: Changing the dose of warfarin to follow the hospital protocol without speaking to the consultant is not appropriate as it goes against the principle of open communication and collaboration between doctors.

Option F: Asking the pharmacist to contact the consultant to discuss the dosage directly with her is not appropriate as it may cause confusion and miscommunication. The doctor should be the one communicating with the consultant regarding the prescription.

Option H: Asking the consultant to explain why she asked you to prescribe a high dose of warfarin is not inappropriate, but it may not be the most effective way to address the issue at hand, which is to follow the hospital protocol.

20

The three most appropriate actions to take in this situation would be Option A, Option C, and Option F.

Option A: Reassure the patient that you were paying attention during her consultation. Rationale: It is important to acknowledge the patient's concerns and reassure them that their consultation was not disregarded. By acknowledging their feelings and reassuring them, the patient may feel heard and be willing to listen to an explanation.

Option C: Explain to the patient that you were looking at her observations on your hospital phone. Rationale: It is important to explain to the patient the reason for looking at your phone during the consultation. By providing an explanation, it can help the patient understand why you were looking at your phone and may alleviate any misunderstandings.

Option F: Explain to the patient that doctors frequently have to check information on their hospital phones. Rationale: By providing the patient with general information about the use of hospital phones, it can help them understand why doctors may use them during consultations. This may also help prevent similar situations from occurring in the future.

Option B: Apologize to the patient for appearing rude during her consultation, Option D: Tell the patient that you were using your hospital phone so that she could receive the best possible care, Option E: Advise the patient that in the future you will tell patients why you are checking your phone during consultations, and Option G: Show the patient her observations that you were looking at on your hospital phone are not the most appropriate actions to take.

Option B: Apologize to the patient for appearing rude during her consultation. Rationale: While an apology may seem like a good solution, it may not be appropriate in this situation because you were not intentionally being rude. Apologizing could also imply that you did something wrong.

Option D: Tell the patient that you were using your hospital phone so that she could receive the best possible care. Rationale: While it is important to prioritize patient care, telling the patient that you were using your phone for their benefit may not address their concerns and could be perceived as dismissive.

Option E: Advise the patient that in the future you will tell patients why you are checking your phone during consultations. Rationale: While it is important to communicate with patients during consultations, making a blanket statement like this could be perceived as insincere and may not address the patient's specific concerns.

Option G: Show the patient her observations that you were looking at on your hospital phone. Rationale: While it is important to be transparent with patients, showing the patient their observations may not address their concerns and could be perceived as dismissive. It is important to acknowledge their feelings and explain why you were looking at your phone during the consultation.

21

The three most appropriate actions to take in this situation are: Option A, Option B, and Option D.

Option A: “I'm sorry but there is an emergency case that takes priority.”

Rationale: This response shows empathy towards the patient and their relatives while providing a clear and concise explanation of why the operation has been cancelled. It is important to acknowledge the patient's disappointment, but also to explain that the hospital must prioritize emergency cases, as this is a matter of patient safety. By providing a clear and understandable explanation, it may help to defuse the situation and prevent further anger or frustration from the patient and their relatives.

Option B: “I can see you are upset. I know this must be disappointing.”

Rationale: This response shows empathy towards the patient and their relatives and acknowledges their feelings about the situation. It is important to validate the patient's emotions and show that you understand why they are upset. By acknowledging their disappointment, it may help to defuse the situation and prevent further anger or frustration from the patient and their relatives.

Option D: “Is there anything else I can do to help?”

Rationale: This response shows a willingness to assist the patient and their relatives in any way possible. It is important to show that you are there to support them and address their concerns. By offering to help, it may help to defuse the situation and prevent further anger or frustration from the patient and their relatives.

Option E: “The registrar/specialty trainee has made the decision; I can not do anything about it I’m afraid.”

Rationale: This response is not appropriate because it may be perceived as dismissive or unhelpful. It is important to show empathy and support towards the patient and their relatives, rather than simply deferring to the decision of a colleague.

Option F: “You can wait to discuss this further with the registrar/specialty trainee if you would prefer?”

Rationale: This response is not appropriate because it may not address the patient and their relatives' concerns. It is important to show empathy and support towards the patient and their relatives, rather than simply suggesting that they wait to discuss the issue with someone else.

Option G: “I understand your frustration but getting angry will not help.”

Rationale: This response is not appropriate because it may be perceived as confrontational or dismissive of the patient and their relatives' emotions. It is important to show empathy and support towards the patient and their relatives, rather than dismissing their concerns.

Option H: “There is no need to shout at me, I can not change anything.”

Rationale: This response is not appropriate because it may be perceived as defensive or confrontational. It is important to show empathy and support towards the patient and their relatives, rather than becoming defensive or dismissive of their concerns.

22

The most appropriate actions to take in this situation are Options A, F, and G.

Option A: Suggest to your FY2 colleague that he requests feedback from someone else instead of his girlfriend. Asking for feedback from someone who has a personal relationship with the individual requesting the feedback can be viewed as unprofessional and may not provide constructive criticism. Suggesting that your colleague requests feedback from another person would be the best course of action in this situation.

Option F: Explain the benefits of receiving constructive feedback to your colleague. It may be helpful to explain the importance of receiving constructive feedback and how it can help your colleague improve their skills as a doctor. This will encourage your colleague to seek feedback from more appropriate sources and improve their learning portfolio.

Option G: Arrange to discuss the situation with your educational supervisor. Reporting this incident to your educational supervisor would be appropriate as it would allow for further guidance and support to be provided. It is important to ensure that the behaviour of colleagues is professional and meets the standards required for the medical profession.

Option B: Contact his girlfriend, recommending that she does not complete the feedback form, is not an appropriate action to take as it could be viewed as interfering in their personal relationship.

Option C: Ask your FY2 colleague if he thinks his girlfriend will respond to his request for feedback, may not be a useful course of action as your colleague may not be able to judge their girlfriend's objectivity in providing feedback.

Option D: Reporting this incident to your FY2 colleague’s educational supervisor may not be necessary if the situation can be resolved by suggesting alternative sources for feedback and explaining the benefits of receiving constructive feedback.

Option E: Informing your FY2 colleague that his behaviour is unprofessional may not be a helpful course of action as it may cause unnecessary tension between colleagues and could potentially harm their working relationship.

Option H: Offering to provide feedback to your FY2 colleague yourself may not be appropriate as it may not be feasible for you to provide constructive feedback that is of a higher standard than his colleagues.

23

The three most appropriate actions to take in this situation are:

Option A: “The registrar is not happy that I have missed some bedside teaching sessions.” Option C: “I would like us to split the work more evenly in future so that we both can meet all of our learning objectives.” Option G: “I understand you do not like going to theatre, but it is an important part of the job.”

Rationale:

Option A: This statement acknowledges the issue raised by the registrar/specialty trainee and shows that you are aware of the impact of spending more time in theatre. This also indicates that you are taking responsibility for your actions.

Option C: This option is a proactive approach to ensure that both you and your colleague can meet your learning objectives. By suggesting to split the work more evenly, it shows that you are willing to help your colleague and are also concerned about your own learning.

Option G: This option acknowledges your colleague's feelings but also emphasizes the importance of going to theatre. As an FY1 doctor, it is essential to be exposed to various aspects of the job, including theatre, to gain the necessary experience and skills.

Options B, D, E, F, and H are not appropriate for this situation. Option B makes it seem like you are making an excuse for missing the bedside teaching sessions, which is not professional. Option D may come across as condescending to your colleague and does not address the root of the problem. Option E is not your responsibility to suggest that your colleague talks to their supervisor about their issue. Option F places the blame on your colleague and does not offer a solution. Option H is not professional and may create tension between you and your colleague.

24

The three most appropriate actions to take in this situation are: Option A, Option E, and Option F.

Option A: “Is there a reason why have you been refusing to take your medication?” As a doctor, it is essential to understand the patient's reasons for not taking their medication, as there may be underlying issues that need to be addressed. Asking the patient why they are refusing medication will help to identify any concerns they may have and potentially find a solution.

Option E: “Are there any questions that you would like to ask me about your medication?" Encouraging the patient to ask questions about their medication will provide an opportunity for them to express any concerns they may have. It also demonstrates that you are willing to listen and help to address their concerns.

Option F: “I'm sorry, Dr Lee should not have spoken to you like that.” It is unprofessional and inappropriate for a doctor to make such comments to a patient. By apologizing on behalf of your colleague, you can help to alleviate any distress caused to the patient and maintain a professional relationship with them.

Option B: "I really think that you should listen to the advice that Dr Lee is giving you.” This statement is not appropriate as it may come across as dismissive of the patient's concerns and dismissive of the fact that they have refused medication.

Option C: “Do you not want to get any better?” This statement is not appropriate as it may come across as accusatory and judgmental, which could cause the patient to become defensive and not disclose their concerns.

Option D: “Do not worry, there are no short term impacts of you not taking your medication.” While this statement may be true, it does not address the underlying issue of why the patient is refusing medication. It may also give the impression that it is not important to take medication, which could cause further problems in the future.

Option G: “Do not get upset, if you want to talk to me, I am here to help.” While this statement is well-intentioned, it may come across as dismissive of the patient's concerns and suggest that they are being overly emotional, which could cause them to become defensive.

25

The three most appropriate actions to take in this situation are:

Option B: Speak to your clinical supervisor* about Dr Lee’s behaviour towards Patricia. This option is appropriate because Dr Lee's behaviour towards Patricia is unprofessional and may be considered as a breach of patient rights. By informing the clinical supervisor, the appropriate measures can be taken to address the situation and prevent it from recurring. Additionally, it is crucial to ensure that the patients' rights and dignity are upheld, and they are treated with respect and compassion.

Option E: Suggest that Dr Lee speaks to the consultant about his concerns. This option is appropriate because Dr Lee's behaviour may be due to the pressure he is facing to discharge patients quickly. By suggesting he speaks to the consultant, he can raise his concerns about how the pressure to discharge patients quickly is affecting his relationship with patients. The consultant may provide support and guidance on how to manage the workload effectively without compromising the patients' care.

Option F: Advise Dr Lee apologizes to Patricia for the way he spoke to her. This option is appropriate because Dr Lee's comment was unprofessional and insensitive. Apologizing to Patricia would show that he recognizes his mistake, and it may help to restore her confidence and trust in him. It is also crucial to ensure that the patients' emotional well-being is maintained while in the hospital.

Rationale: Option A: Suggest to Dr Lee that he takes a break before finishing the ward round. While taking a break may be helpful in reducing stress levels, it does not address the root cause of the problem. Dr Lee's behaviour is unprofessional, and patients' rights must be upheld at all times. Therefore, taking a break is not an appropriate response to the situation.

Option C: Ask your colleagues on the ward to monitor Dr Lee's behaviour with patients. While it is important to monitor Dr Lee's behaviour with patients, it is not an appropriate response to the current situation. Monitoring his behaviour may be necessary in the future, but the immediate concern is addressing the unprofessional behaviour towards Patricia.

Option D: Explain to Dr Lee that you understand that he is under a lot of pressure. While acknowledging Dr Lee's pressure may be helpful, it does not address the unprofessional behaviour towards Patricia. Moreover, it may come across as being dismissive of Patricia's feelings and concerns, which is not appropriate.

Option G: Suggest to Dr Lee that he stops trying to discharge patients early and focuses on maintaining positive relationships. While maintaining positive relationships with patients is important, it is not always possible to avoid early discharges. Additionally, this response does not address the current situation, which is the unprofessional behaviour towards Patricia.

Option H: Ask Dr Lee if there is anything that you can do to support him. While offering support is important, it does not address the root cause of the problem. Additionally, it may be inappropriate to offer support to someone who has behaved unprofessionally towards a patient. Therefore, it is not an appropriate response to the situation.

26

Option A: “I am sorry that I offended you with my comment, but making a complaint will not help your daughter.” Rationale: This option shows empathy towards the parents and acknowledges their feelings of being offended. It also attempts to redirect their focus to their daughter's health rather than making a complaint, which may not help their daughter's current condition.

Option D: “I understand this situation with your daughter must be difficult for your family.” Rationale: This option shows empathy towards the parents and acknowledges the difficult situation they are going through. It can help to defuse any tension or frustration the parents may be feeling.

Option F: “Please give me the opportunity to explain the facts about smoking and pulmonary complications.” Rationale: This option attempts to educate the parents about the link between smoking and pulmonary complications in their daughter, which may help them to understand the importance of quitting smoking. It also shows that the doctor is knowledgeable and can provide helpful information to the parents.

Overall, the most appropriate actions to take in this situation are those that show empathy towards the parents, acknowledge their feelings, and provide education about the link between smoking and pulmonary complications. Options A, D, and F best reflect these principles.

27

The three most appropriate actions to take in this situation are: Option B, Option E, and Option H.

Option B: Explain the situation to your clinical supervisor, seeking advice on how to proceed It is important to seek guidance from a senior colleague to handle this situation appropriately. This will help ensure that the situation is managed appropriately and that the best possible care is provided to the patient.

Option E: Speak to the senior nurse involved, obtaining further details about the incident It is important to obtain more information about what happened from the senior nurse involved in the incident. This will help clarify what happened and help determine the appropriate next steps.

Option H: Fill in a critical incident form, documenting the incident It is important to document the incident in a critical incident form to ensure that it is properly investigated and that any necessary changes are made to prevent similar incidents from occurring in the future.

Rationale: Option A: Inform Hussein's clinical supervisor of the allegations that he has made about the senior nurse While it is important to ensure that any allegations made are properly investigated, it may not be appropriate to immediately inform Hussein's clinical supervisor without first gathering more information and seeking advice from a senior colleague.

Option C: Advise Hussein of the severity of his allegations, ensuring that he is aware of what he is saying While it is important to address any concerns or allegations made by staff, it may not be appropriate to immediately confront Hussein without first gathering more information and seeking guidance from a senior colleague.

Option D: Inform the senior nurse that Hussein told you that she was lying about the patient with chest pain This option may cause unnecessary conflict and could make it difficult to manage the situation appropriately. It is important to gather more information and seek guidance from a senior colleague before taking any action.

Option F: Explore with the other nurses whether they overheard the telephone call between Hussein and the senior nurse This option may not be appropriate, as it could create tension and mistrust among the nursing staff. It is important to gather more information and seek guidance from a senior colleague before taking any action.

Option G: Suggest to Hussein that he talks to the senior nurse about the incident While it is important to encourage staff to communicate and resolve any issues, it may not be appropriate to suggest this without first gathering more information and seeking guidance from a senior colleague.

28

The THREE most appropriate actions to take in this situation are Option B, Option C, and Option F.

Option B: Suggest to Abigail that she takes some time off work until she feels less stressed. This is an appropriate action because if Abigail is feeling stressed to the point where it is negatively affecting her mental health, it may be necessary for her to take some time off work to recuperate. This will also ensure that patient safety is not compromised due to her being too stressed to perform her duties adequately.

Option C: Ask Abigail if she has thought about seeing her GP. This is an appropriate action because it is important for Abigail to seek professional medical advice if she is experiencing mental health issues. The GP can provide her with appropriate support and guidance and may also suggest further referral to a mental health specialist if required.

Option F: Advise Abigail to speak to her educational supervisor about how she is feeling. This is an appropriate action because Abigail's educational supervisor can provide her with support and guidance on how to manage her workload and stress levels. The supervisor may also suggest appropriate support services available to her within the hospital or externally.

Option A is not appropriate as Abigail may not feel comfortable discussing her mental health outside of work with a colleague.

Option D is not appropriate as it may trivialize Abigail's feelings of stress and anxiety.

Option E may not be practical as reducing Abigail's workload may not be possible or practical, particularly in a busy surgical ward.

Option G is not appropriate as it does not offer any specific support or guidance to Abigail on how to manage her stress levels.

Option H is not appropriate as it may not provide the necessary support and guidance to Abigail to manage her stress levels effectively.

29

The three most appropriate actions to take in this situation are Option A, Option E, and Option G.

Option A: Ask Abigail if she can help you to prioritise the most important jobs. Rationale: Asking Abigail to help prioritise the most important jobs will help to ensure that essential tasks are completed before she leaves. This will also demonstrate empathy and support for her situation.

Option E: Suggest to Abigail that she takes a ten-minute break and then sees if she can continue working. Rationale: Suggesting that Abigail takes a short break may help her to regain her composure and allow her to continue working. It will also demonstrate empathy and support for her situation.

Option G: Ask Abigail how she intends to make sure that her jobs are completed if she leaves now. Rationale: This option will help to ensure that Abigail's outstanding tasks are completed. It will also allow for a discussion about how to ensure the continuity of patient care and demonstrate a proactive approach to the situation.

Option B, Option C, Option D, Option F, and Option H are not appropriate actions to take in this situation.

Option B: Tell Abigail that you need her to finish her jobs before she can leave. Rationale: This option is not appropriate as it may cause further stress and upset for Abigail. It also does not take into account her mental health concerns.

Option C: Advise Abigail that you have your own jobs to finish and cannot complete her jobs if she leaves. Rationale: This option is not appropriate as it may lead to essential tasks being left incomplete, which could compromise patient care.

Option D: Ask Abigail if she is sure that she cannot carry on working. Rationale: This option is not appropriate as Abigail has already expressed that she is feeling stressed and unable to continue working. It also does not provide any practical solutions to the situation.

Option F: Inform Abigail that she will need to tell other ward staff if she intends to leave now. Rationale: This option is not appropriate as it does not address the outstanding jobs on Abigail's list or provide any practical solutions to ensure continuity of patient care.

Option H: Ask Abigail if she would feel better if she swapped some jobs with you. Rationale: This option is not appropriate as it does not address the outstanding jobs on Abigail's list or provide any practical solutions to ensure continuity of patient care.

30

The most appropriate actions to take in this situation are B, E, and F.

Option B: Apologise to Mrs Ratnaike for Haroon's behaviour. It is important to acknowledge Mrs Ratnaike's distress and apologise for the behaviour of a colleague, even if you were not directly involved in the incident. This can help to calm the patient down and begin to rebuild trust.

Option E: Ensure that Mrs Ratnaike is comfortable. As a healthcare professional, your first priority should always be to ensure that your patient is comfortable and receiving appropriate care. This could involve providing pain relief or arranging for further assessment by a more senior doctor.

Option F: Inform your consultant of the incident. It is important to involve a senior member of staff to help manage the situation and provide support. They can also ensure that appropriate action is taken to address the behaviour of the colleague involved, and prevent similar incidents from occurring in the future.

Option A: Telephone Haroon, explaining that he made Mrs Ratnaike cry, and Option G: The next time he is at work, inform Haroon that he made Mrs Ratnaike cry, could be appropriate actions to take, but only after consulting with a senior colleague. It may be more appropriate to involve a senior member of staff first, who can then have a conversation with Haroon to address his behaviour.

Option C: Advise Mrs Ratnaike of the hospital's complaints procedure, and Option D: Explore with your FY1 doctor colleagues whether they have ever witnessed similar behaviour from Haroon, are not appropriate actions to take in this situation. Mrs Ratnaike is already upset and it may not be helpful to discuss complaints procedures at this stage. Additionally, it is not appropriate to discuss a colleague's behaviour with other colleagues before taking appropriate steps to address the situation.

31

The three most appropriate actions to take in this situation are:

Option B: Arrange a private conversation with your FY1 colleague to explain the impact of his lateness.

This option would be a good first step in addressing the issue. A private conversation with the colleague would allow you to discuss the impact of his lateness and explore any reasons why he might be consistently late. This conversation could also be an opportunity to provide guidance on how to manage time better and suggest strategies that could help him arrive on time.

Option F: Suggest to the team that you start the morning handover on time, even if someone is missing.

Starting the handover on time, regardless of whether everyone is present, is an effective way to manage the team's time and ensure that they finish their work on time. It is essential to communicate to the team that this is a priority, and everyone needs to be punctual. This can help ensure that the team is not delayed by one person and can start their work on time.

Option G: Ask your FY1 colleague if he is having any difficulties getting into work on time.

This option allows you to explore any potential issues that may be contributing to the colleague's lateness. It is essential to approach this conversation sensitively, as there may be a variety of reasons why the colleague is consistently late. Some potential reasons could be transport issues or personal problems. By offering help and support, you may be able to identify ways to address the problem.

Rationale for each answer:

Option A: Reporting the colleague to the clinical supervisor should only be considered as a last resort after other options have been exhausted. It may be better to try to resolve the issue at a lower level before escalating it.

Option C: Monitoring the colleague's behavior may be necessary, but it may not resolve the issue. It is essential to take action if the behavior continues.

Option D: Discussing the situation with other colleagues on the ward may not be appropriate as it may lead to gossip and speculation.

Option E: Informing colleagues that one of the colleagues is consistently late may lead to a toxic work environment and cause unnecessary tension among colleagues.

Option H: Asking the colleague if he is clear on what time the morning handover and ward round starts may be helpful, but it does not address the issue of consistent lateness.

32

The most appropriate actions to take in this situation are A, B, and E.

A) Consider how you can learn from this to inform how you behave in future situations: It is important to reflect on the situation and think about what could have been done differently. This will help to avoid making similar mistakes in the future and to improve communication with patients.

B) Tell Kathryn that it must be difficult to feel that she is being judged by others: This shows empathy and understanding towards Kathryn's feelings. It is important to acknowledge her emotions and to show that you care about how she feels.

E) Tell Kathryn that you can ask one of your colleagues to treat her if she would prefer: It is important to offer an alternative healthcare professional to Kathryn if she does not feel comfortable with being treated by you. This shows that you respect her wishes and that you want her to receive the best care possible.

Options C, D, F, G, and H are not appropriate actions to take in this situation.

C) Apologise to Kathryn and Tristan, explaining that you did not mean to cause any offence: While it is important to apologise for any misunderstanding or offence caused, this option does not address Kathryn's concerns and may not resolve the situation.

D) Make a joke about the situation in an attempt to calm the situation down: This is not appropriate as it could be perceived as insensitive and dismissive of Kathryn's feelings.

F) Explain to Kathryn that if she would like to be treated now, she will need to be treated by you: This is not appropriate as it may cause further upset and distress to Kathryn. It is important to offer her an alternative healthcare professional if she does not feel comfortable being treated by you.

G) Ask Kathryn if she would like you to tell your colleagues on the department that Tristan is her boyfriend to prevent a similar mistake: This is not appropriate as it is not respectful towards Kathryn and may be seen as an attempt to shift the blame onto her.

H) Explain to Kathryn that you were just trying to ensure that she has the necessary care at home: This is not appropriate as it does not address Kathryn's concerns and may not resolve the situation.

33

The three most appropriate actions to take in this situation are:

Option A: Inform the nurse on the ward that you are taking Clare to a quiet place to calm her down This is an important first step to take as Clare is visibly distressed and needs support. Moving her to a quieter area can help calm her down and allow her to speak more freely about what happened.

Option B: Report the senior doctor to their supervising consultant It is important to report the senior doctor's behavior to their supervising consultant as it is not acceptable for a senior doctor to shout at a junior doctor in this manner. This behavior can be detrimental to Clare's mental wellbeing and can also negatively impact patient care.

Option D: Suggest that Clare discusses her concerns with her educational supervisor This is an appropriate action as the educational supervisor can provide support and advice on how to deal with this situation. Additionally, the educational supervisor can provide guidance on how to prevent similar situations from occurring in the future.

Rationale: Option A: It is important to provide immediate support to Clare to help her calm down and provide a safe space to talk about what happened.

Option B: Reporting the senior doctor's behavior is important as it sets an example that such behavior is not acceptable in the workplace. It is also important to report the behavior to ensure that it does not happen to other junior doctors in the future.

Option D: The educational supervisor can provide support and guidance to Clare on how to handle this situation, and can also provide advice on how to prevent similar situations from occurring in the future. The educational supervisor can also ensure that Clare's mental wellbeing is being taken care of.

34

The THREE most appropriate actions to take in this situation are:

Option C: Empathise with Pat that it must be difficult for her, having to have so many tests. Option D: Explain to Pat that the tests are important, so that the team can give her the best possible care. Option G: Ask Pat whether she understands why the CT scan is required and if she has any questions.

Rationale:

Option A is not appropriate because it does not address the patient's concerns or provide any explanation for the need for the test. Patients have the right to refuse treatment, but it is important to explain the potential benefits and risks of any tests or treatments.

Option B is not appropriate because it is not true. Patients have the right to refuse treatment, and forcing a patient to undergo a test or treatment against their wishes is not ethical.

Option C is appropriate because it acknowledges Pat's feelings and shows empathy. It is important to listen to patients and understand their concerns, which can help to build trust and improve communication.

Option D is appropriate because it provides Pat with information about the importance of the test, which can help her to make an informed decision. It is important to explain the potential benefits and risks of any tests or treatments, so that patients can make an informed decision.

Option E is not appropriate because it does not address Pat's concerns or provide any explanation for the need for the test. It may be helpful to offer emotional support, but it is important to address the patient's concerns first.

Option F is not appropriate because it does not address Pat's concerns or provide any explanation for the need for the test. It may be helpful to offer assistance, but it is important to address the patient's concerns first.

Option G is appropriate because it encourages Pat to ask questions and seek clarification. It is important to ensure that patients understand the reason for any tests or treatments, as this can improve adherence and patient satisfaction.

Option H is not appropriate because delaying further tests could compromise Pat's care. It is important to address any concerns and provide information, so that patients can make an informed decision about their care.

35

The THREE most appropriate actions to take in this situation are:

Option B: Try and find out more details about the second daughter's concerns. Option E: Ask Occupational Therapy* to assess the patient. Option F: Ask the first daughter if she has any concerns about her mother being able to cope at home.

Rationale:

Option B is important because it is necessary to obtain more information about the second daughter's concerns. This will allow the doctor to understand why she feels that her mother is not coping at home and whether there are any real concerns that need to be addressed before discharge.

Option E is important because it is necessary to assess whether the patient is able to cope at home. Occupational Therapy can evaluate the patient's ability to perform activities of daily living and recommend any necessary adaptations to the home environment.

Option F is important because it is necessary to obtain the first daughter's perspective on her mother's ability to cope at home. She may have important insights into her mother's current situation and any additional support that may be required.

Option A should not be selected because it does not take into account the concerns of the second daughter or the need to assess the patient's ability to cope at home.

Option C should not be selected because it is not appropriate to involve the patient in any family conflicts or disagreements.

Option D should not be selected because it is not appropriate to discuss the patient's discharge with the second daughter without the patient's consent.

Option G should not be selected because it does not address the concerns of the second daughter or the need to assess the patient's ability to cope at home.

Option H should not be selected because it places the responsibility on the patient to make a decision about her discharge, without taking into account the concerns of her family members or her ability to cope at home.

36

The THREE most appropriate actions to take in this situation are:

Option A: Speak to Mr Hamilton, with the nurse in charge present, to try to explore his concerns further. Option B: Ask the specialty trainee to speak with Mr Hamilton upon his return from clinic. Option H: Acknowledge Mr Hamilton's frustrations.

Rationale:

Option A: It is important to communicate with Mr Hamilton and understand his concerns about his son's illness. This will help to alleviate his anxiety and frustration and build trust between the healthcare team and the patient's family. Having the nurse present can help to ensure a safe environment and support the conversation.

Option B: It is appropriate to involve the specialty trainee in the conversation with Mr Hamilton as they have more experience and knowledge than an FY1 doctor. The trainee can provide more detailed explanations about the investigation process and answer any medical questions that Mr Hamilton may have.

Option H: It is crucial to acknowledge Mr Hamilton's frustrations and empathize with him. This can help to de-escalate the situation and show that the healthcare team is taking his concerns seriously. It is important to reassure Mr Hamilton that the healthcare team is doing everything possible to diagnose and treat his son's illness.

Option C, D, E, and F are not appropriate actions in this situation. Option C involves waiting until the consultant is back on the ward, which may not be timely or practical. Option D is not appropriate as it can escalate the situation and cause more distress for both Mr Hamilton and Caleb. Option E is confrontational and may escalate the situation further. Option F may not address Mr Hamilton's immediate concerns and may not be helpful in resolving the situation.

Option G is partially appropriate as it informs Mr Hamilton about the investigation process, but it does not address his concerns and may not be sufficient in de-escalating the situation. Therefore, it is not one of the three most appropriate actions to take in this scenario.

37

The most appropriate actions to take in this situation are Options A, B, and E.

Option A: Inform James that he has labelled Mr Rowley's blood test wrong. It is essential to inform James of his mistake so that he can learn from it and avoid making similar errors in the future. By addressing the mistake, the situation can be resolved quickly, and patient safety can be maintained.

Option B: Encourage James to double check the labelling of blood tests in the future. By encouraging James to double-check his work, he can avoid making the same mistake in the future. This approach can help prevent future errors and promote patient safety.

Option E: Suggest to James that he gets someone to check over his labelling of blood tests in future. Getting someone to check James's labelling of blood tests in the future is another way to prevent future errors. Having a colleague review his work can catch any mistakes before they become significant problems.

Options C, D, F, G, and H are not appropriate actions to take in this situation.

Option C: Explain to James that the laboratory has stated that they have no blood tests for a Mr Rowley. This option may create a confrontational situation, and it may make James defensive. It is better to address the situation directly with James rather than making him feel like he is being blamed by a third party.

Option D: Ask James if he remembers labelling Mr Rowley's blood tests. This question may also make James defensive, and it does not provide a direct solution to the problem at hand. It is better to inform James of the mistake and work on preventative measures for the future.

Option F: Tell James that you will not ask him to complete blood tests for your patients again. This option is an extreme measure and may not be necessary. James may learn from his mistake and be more cautious in the future.

Option G: Inform James that Mr Rowley will not be very happy with him. This statement is not necessary and may create unnecessary stress and tension for James. It is best to address the situation directly with James and avoid involving patients in internal hospital matters.

Option H: Tell James that he has wasted the laboratory's time. This statement is not constructive and may create tension between James and the laboratory. It is better to focus on addressing the mistake and implementing preventative measures for the future.

38

The THREE most appropriate actions to take in this situation are: Option A, Option B, and Option F.

Option A: Request the scan yourself As the patient requires an urgent scan, it is important to take action to ensure that the scan is requested as soon as possible to avoid any delay in diagnosis or treatment. Therefore, the most appropriate action is to request the scan yourself, ensuring that the patient receives the necessary care in a timely manner.

Option B: Ask Alberto why the scan was not requested It is important to understand why Alberto did not request the scan to prevent similar issues from happening in the future. By asking Alberto why the scan was not requested, you may uncover a valid reason for the delay or identify any issues that need to be addressed.

Option F: Check if any other urgent tasks are incomplete If Alberto has left an urgent task unfinished, it is possible that other tasks are incomplete as well. Therefore, it is important to check if any other urgent tasks are incomplete and to address them as soon as possible to ensure that the patients receive the necessary care.

Option C: Talk to Alberto’s clinical supervisor* about his performance While it is important to ensure that the patient receives the necessary care, speaking to Alberto's clinical supervisor about his performance may be premature at this point. It is important to first address the immediate issue at hand before escalating the situation to a higher authority. It is possible that Alberto may have a valid reason for the delay or that there may be other factors at play that need to be addressed first.

Option D: Tell Alberto he should apologize to the patient for the delay While it is important to acknowledge any delays in care, telling Alberto to apologize to the patient for the delay may not be the most appropriate action at this point. It is important to first address the immediate issue at hand and determine the reason for the delay before discussing any apologies.

Option E: Advise the nurse to discuss this patient with Alberto As the responsibility for requesting the scan lies with the doctors, advising the nurse to discuss the patient with Alberto may not be the most appropriate action. It is important to ensure that the patient receives the necessary care in a timely manner, and the most appropriate action is to request the scan yourself and address any issues with Alberto directly.

Option G: Inform the consultant that Alberto has not requested the scan While it is important to ensure that the patient receives the necessary care, informing the consultant about Alberto's performance may be premature at this point. It is important to first address the immediate issue at hand before escalating the situation to a higher authority. It is possible that Alberto may have a valid reason for the delay or that there may be other factors at play that need to be addressed first.

Option H: Ask your fellow FY1s on the ward if they have had any similar experiences while working with Alberto While it may be helpful to gather information about Alberto's performance, asking fellow FY1s about their experiences with Alberto may not be the most appropriate action at this point. It is important to first address the immediate issue at hand and determine the reason for the delay before discussing any broader issues.

39

The three most appropriate actions to take in this situation are:

Option A: Ask the nurse to ensure that he is mindful of the urgency of a situation when he is communicating in future Option C: Discuss with the nurse why he thought that the situation was urgent Option E: Apologise for confusing the patient as there is nothing wrong

Rationale:

Option A is important because it is necessary to ensure that the nurse understands the importance of communicating accurately and clearly, especially when indicating that a patient is unwell. By asking the nurse to be mindful of the urgency of a situation, it is hoped that he will be more precise when communicating information in the future.

Option C is important because it is necessary to discuss with the nurse why he thought that the situation was urgent. It could be that the nurse is inexperienced or overreacting, and this conversation will help clarify what happened and prevent similar incidents from happening in the future.

Option E is important because it is necessary to apologise to the patient for any distress that the misunderstanding may have caused. Apologising to the patient is essential as it shows that the healthcare team is empathetic and takes the patient's experience seriously.

Option B, D, F, G, and H are not appropriate actions in this situation because they do not address the issue of why the nurse thought the situation was urgent and they do not address the confusion caused to the patient. It is essential to address the root cause of the issue to prevent future similar incidents and to ensure that the patient's experience is not negatively impacted.

40

The three most appropriate actions to take in this situation are B, D, and E.

Option B: Ask the patient to tell you more about this situation and her concerns This is important to gain a deeper understanding of the patient's concerns and perspective. It also shows empathy towards the patient and helps establish rapport, which may ultimately lead to a solution.

Option D: Document the patient’s concerns in the nursing notes Documenting the patient's concerns is important for communication and continuity of care. This also ensures that other healthcare professionals are aware of the patient's concerns and can address them accordingly.

Option E: Inform the nursing staff that the patient is having trouble sleeping because of the noise levels at night Informing the nursing staff of the patient's concerns can help them to address the issue and take appropriate action to reduce the noise levels at night. This also demonstrates advocacy for the patient and shows that their concerns are being taken seriously.

Option A: Offer the patient some general advice on how to sleep better at night While this may be helpful advice in general, it may not address the patient's specific concerns about the noise levels at night. It is important to first address the patient's immediate concerns before offering general advice.

Option C: Explain that telephones are only used at night by nurses for urgent issues that cannot wait until the morning While this is important information, it may not necessarily address the patient's concerns about the noise levels at night. It is important to first address the patient's immediate concerns before explaining hospital policies.

Option F: Arrange for the patient to be given some ear plugs While this may be a solution, it should not be the first option as it does not address the underlying issue of noise levels at night. It is important to first address the patient's immediate concerns before offering solutions that may not be necessary.

Option G: Ask the ward manager about the feasibility of moving the patient to a different bed on the ward While this may be a solution, it is not always feasible and may not be necessary. It is important to first address the patient's immediate concerns before exploring more drastic solutions.

Option H: Offer to prescribe a low dose sleeping tablet While this may be a solution, it should not be the first option as it does not address the underlying issue of noise levels at night. It is important to first address the patient's immediate concerns before offering solutions that may not be necessary. Additionally, prescribing sleeping tablets should be done with caution and only when necessary.

**Answers and rationale for ranking questions:**

41

Ranking:

1. Option A: The health and wellbeing of your colleague
2. Option C: The impact on patient safety if he is working while tired
3. Option D: The impact on the team if he takes time off work
4. Option E: The impact on your workload if he takes time off work
5. Option B: The opportunity for financial gain by working extra shifts if he takes time off work

Rationale:

1. Option A: The health and wellbeing of your colleague is the most important consideration in this situation. It is important to prioritize your colleague's physical and mental health and support him in seeking the appropriate help and resources. This will help him recover and return to work safely and effectively.
2. Option C: The impact on patient safety if he is working while tired is also a critical consideration. Tiredness can impair a doctor's judgment and decision-making abilities, which can lead to medical errors and harm to patients. It is essential to ensure that your colleague is fit to work and not endangering patients.
3. Option D: The impact on the team if he takes time off work is an important consideration, as it may affect the workload and morale of other team members. However, this should not take priority over your colleague's health and safety or patient safety.
4. Option E: The impact on your workload if he takes time off work is a relevant consideration, but it should not be prioritized over the first three options. Your workload can be managed by redistributing tasks and responsibilities among team members, and your colleague's health and safety should not be compromised.
5. Option B: The opportunity for financial gain by working extra shifts if he takes time off work is the least important consideration in this situation. It is not appropriate to pressure a colleague to work when they are not physically or mentally fit to do so, and financial gain should not take precedence over the health and safety of your colleague and patients.

42

1. Option D: “I appreciate you are comfortable here, but unfortunately there is a severely ill patient who will benefit from this room even more so than you” - This is the most important consideration in the management of this situation as it is important to explain to Julia the reason behind the decision to move her to another room. It is crucial to emphasize that the move is not a personal decision, but one based on the needs of a more severely ill patient. This explanation will help Julia understand the rationale behind the decision, and it may ease her concerns.
2. Option B: “We would like to offer a side room to every patient, but there are not enough rooms for every patient to be in a separate room” - This consideration is important because it helps to explain the reason why Julia cannot be accommodated in a side room. It is important to communicate that side rooms are limited and they have to be allocated based on clinical need. This explanation can help Julia understand that it is not a personal decision, but rather a logistical one.
3. Option C: “We must prioritise side rooms based on clinical need and you are not considered a priority on this basis” - This consideration is also important as it explains the allocation process for side rooms. However, it is not as important as option B because it may come across as dismissive and insensitive to Julia’s concerns.
4. Option A: “Unfortunately, you do not get to decide which room you stay in” - This consideration may be perceived as confrontational, and it may make Julia feel disempowered. It is important to acknowledge her concerns and provide a clear explanation of why the decision was made, rather than dismissing her concerns.
5. Option E: “If you have a problem with the decision, you should speak to the nurse in charge” - This consideration is the least important in this situation because Julia has already expressed her concerns to the doctor. It is important to address her concerns directly and provide an explanation rather than deflecting her concerns to another member of staff.

43

1. Option D: Apologize to Mr Rayner and his son for the incident, explaining mistakes such as this are extremely rare.

The most important consideration in this situation is to show empathy towards the patient and their family. As the doctor responsible for Mr Rayner's care, it is important to take responsibility for the mistake and apologize for any inconvenience caused. By acknowledging the mistake, and explaining that it is rare, it may help to alleviate any anxiety or anger that Mr Rayner and his son may be experiencing.

1. Option A: Reassure Mr Rayner and his son that a full investigation into this incident will take place.

It is important to reassure the patient and their family that the incident will be investigated thoroughly, to prevent a similar mistake from happening in the future. However, this should not be the first response, as it may come across as defensive and not empathetic to their concerns.

1. Option C: Explain to Mr Rayner and his son that you will ask that they are kept up to date with any news of the incident.

Keeping the patient and their family up to date is important, but it should not be the first response. It is more important to apologize and show empathy first, before discussing any further actions.

1. Option E: Offer to ask the nurse in charge of the ward to come and speak to Mr Rayner and his son later.

It is important to take responsibility for the mistake and address the concerns of the patient and their family as the doctor in charge of Mr Rayner's care. While it is appropriate to involve the nurse in charge of the ward in the investigation and resolution of the issue, it is not appropriate to delegate the responsibility of addressing the concerns of the patient and their family to someone else.

1. Option B: Explain to Mr Rayner and his son that you are unable to disclose who booked the transportation.

This option is not appropriate, as it may come across as defensive and unempathetic to the concerns of the patient and their family. As the doctor responsible for Mr Rayner's care, it is important to take responsibility for the mistake and address their concerns.

44

Ranking:

1. Option A: “I will ask Dr Edmund to come back and answer your questions.”
2. Option B: “I will make sure that I understand all the important details about your management before Dr Edmund leaves.”
3. Option D: “Is there something in particular you want to discuss about your management?”
4. Option E: “Don’t worry, I am clear what she wants me to do next.”
5. Option C: “She does speak very quickly, doesn’t she?”

Rationale:

1. Option A: “I will ask Dr Edmund to come back and answer your questions.” This is the most important consideration in this situation. The patient has expressed confusion and concern about their care, and it is important that they have their questions answered by the appropriate medical professional. As an FY1 doctor, it is important to prioritize the patient's needs and ensure that they receive adequate care. Asking Dr Edmund to return and answer the patient's questions is the best way to address their concerns and ensure their safety.
2. Option B: “I will make sure that I understand all the important details about your management before Dr Edmund leaves.” It is important to understand the details of the patient's management to ensure that they receive proper care. As an FY1 doctor, it is important to be familiar with the patient's case and to ask questions if something is unclear. This will help to prevent misunderstandings and ensure that the patient receives appropriate care.
3. Option D: “Is there something in particular you want to discuss about your management?” It is important to address any specific concerns or questions that the patient may have. As an FY1 doctor, it is important to take the time to listen to the patient and address their concerns to ensure that they feel comfortable with their care. This can help to build a better doctor-patient relationship and improve patient outcomes.
4. Option E: “Don’t worry, I am clear what she wants me to do next.” While it is important to understand the next steps in the patient's care, it is not enough to simply understand what is being asked of you. As an FY1 doctor, it is important to ensure that the patient understands their care and is comfortable with it. This requires addressing any concerns or questions that they may have and ensuring that they have a clear understanding of their management.
5. Option C: “She does speak very quickly, doesn’t she?” While it may be true that Dr Edmund speaks quickly, this consideration is the least important in this situation. As an FY1 doctor, it is important to focus on the patient's needs and concerns and ensure that they receive adequate care. Blaming the situation on the speed of Dr Edmund's speech does not address the patient's concerns and is not productive in resolving the situation.

45

1. Option E: Continue managing Mr Cox yourself, following hospital guidelines for managing acute coronary syndrome.

The most important consideration in this scenario is to ensure that Mr Cox's condition is appropriately managed as quickly as possible. As a FY1 doctor, it is essential to have the skills and knowledge to provide emergency management of acute coronary syndrome until specialist support is available. Therefore, the immediate priority should be to assess and stabilize the patient's condition by initiating appropriate treatment and investigations. This option should be the top priority and should not be delayed while waiting for assistance from others.

1. Option A: Contact Lynda again, reiterating the urgency for her to assess Mr Cox immediately.

The specialty trainee should be informed again of the severity of the situation and the urgency to attend. It is important to ensure that the correct protocol is followed and specialist opinion is obtained as soon as possible. If the specialist trainee cannot attend, it is necessary to find an alternative person from the cardiology team to see Mr Cox.

1. Option B: Contact the cardiology consultant, asking him to review Mr Cox since Lynda is too busy.

If the cardiology trainee is unavailable, the next option is to contact the consultant responsible for the cardiology team, and ask him or her to review Mr Cox as soon as possible. This is particularly important if Mr Cox's condition deteriorates, or if there is a significant delay in obtaining specialist input.

1. Option D: Contact a senior colleague from your own team, seeking support in managing the situation.

If the trainee is unavailable and the consultant is not able to attend, then the next option is to seek support from a senior colleague from the same team. However, this option should be considered only after the above options have been exhausted, and the patient's condition is still unstable.

1. Option C: Document in Mr Cox's notes Lynda's refusal to attend.

It is important to document any refusal or delay in specialist input for future reference. However, this option should not be a priority in the immediate management of Mr Cox's condition. Instead, the priority should be to provide appropriate care and seek specialist input as soon as possible.

46

1. Option B: Explain to the mother that you need the patient to answer the questions.

This is the most important consideration because it is essential to obtain accurate information from the patient to make an appropriate diagnosis and provide appropriate treatment. The mother's interference could affect the accuracy of the history and may result in mismanagement of the patient. It is the responsibility of the doctor to ensure that the patient is heard and understood. The doctor should politely explain to the mother that it is important for the patient to answer the questions to understand her symptoms and provide the best possible care.

1. Option C: Ask the mother to leave the cubicle for a moment while you ask the patient some questions.

If the mother's interference continues despite explanation, the doctor should politely ask her to leave the cubicle for a moment. This will allow the doctor to speak to the patient alone and obtain accurate information about her symptoms. However, it is important to ensure that the patient is comfortable with the idea of being alone with the doctor, and her consent should be obtained before proceeding with this option.

1. Option D: Ask the patient if she wants her mother to remain in the cubicle while you continue to ask her some questions.

This option is less important than Option B and Option C. Still, if the patient is uncomfortable with her mother leaving the cubicle, the doctor can ask her if she is comfortable with her mother remaining in the cubicle but remaining quiet while the doctor asks her some questions. However, it is essential to ensure that the mother does not interfere in any way, and the doctor can politely remind her to remain quiet if necessary.

1. Option E: Inform the mother that she can stay in the cubicle but will need to remain quiet.

This option is less important than Option B, C, and D. Still, if the mother insists on remaining in the cubicle and the patient is comfortable with her mother being present, the doctor can inform the mother that she can stay in the cubicle but needs to remain quiet. However, it is important to ensure that the mother does not interfere in any way, and the doctor can politely remind her to remain quiet if necessary.

1. Option A: Let the mother continue to answer the questions.

This option is the least important because it may compromise the accuracy of the history and may result in mismanagement of the patient. The doctor must obtain accurate information from the patient to make an appropriate diagnosis and provide appropriate treatment. The mother's interference could affect the accuracy of the history, and the doctor should not let the mother continue to answer the questions. The doctor should politely explain the importance of obtaining information from the patient to the mother and redirect the conversation to the patient.

47

Ranking:

1. Option D: “It is important we both take our regulatory breaks throughout the day.”
2. Option B: “Can I take my 30 minute break first so that I can clerk the patients safely and effectively.”
3. Option A: “Dr Lough, I’m sorry I am going on my break now. I'll be back in 30 minutes.”
4. Option E: “If you allow me to take a quick 5minute break, I can then quickly help with the rest of the patients.”
5. Option C: “I don't think it is appropriate for you to teach others to avoid taking breaks during their shift.”

Rationale:

Option D is the most important because it emphasizes the importance of taking regulatory breaks for both the doctor and the patient's safety. It is important for doctors to take breaks to avoid burnout and ensure that they are well-rested and able to provide effective care.

Option B is the second most important because it recognizes the need to prioritize patient safety and effectiveness in care by taking a break to refresh and recharge. This approach ensures that the patients are well-catered for and that they receive the necessary care that meets their needs.

Option A is the third most important because it demonstrates assertiveness and professionalism in adhering to the hospital policy on break times, while still acknowledging the need for the patients to be attended to.

Option E is the fourth most important because it offers a temporary solution to the problem of patients waiting while still emphasizing the need for a break.

Option C is the least important because it does not provide a solution to the problem at hand and could potentially escalate the situation further. It also does not prioritize patient care and safety.

48

1. Option D: Discuss the situation directly with Lara's clinical supervisor* The most important consideration in this situation is to ensure patient safety and uphold professional boundaries. If Lara is not willing to listen to your advice, it is important to escalate the situation to a higher authority to ensure that appropriate action is taken to prevent any potential harm to the patient. The clinical supervisor is in a position of authority and responsibility to manage Lara's conduct, and to ensure that she is adhering to the professional standards and ethical principles expected of a doctor. They can also provide guidance and support to Lara on appropriate professional conduct in such situations.
2. Option A: Ask to speak to Lara in private to reiterate that her behaviour is unprofessional It is important to communicate with Lara privately and remind her of her professional boundaries and the potential consequences of her actions. As a colleague, you have a duty to ensure that Lara understands the seriousness of the situation and the importance of maintaining appropriate professional conduct. However, if Lara is not willing to listen to your advice or change her behavior, further action may be necessary.
3. Option B: Advise Lara to tell the patient that she cannot go out for dinner with him It is important to advise Lara to avoid any situations that could lead to a breach of professional boundaries, such as accepting an invitation for dinner from a patient. Suggesting to Lara to tell the patient that she cannot go out for dinner with him is an appropriate response to this situation. However, this option is ranked lower in importance than Option A and D because it relies on Lara to take appropriate action and may not necessarily prevent future breaches of professional conduct.
4. Option E: Ask Lara if she has previously arranged to meet up with patients outside of work This option may provide useful information about Lara's past behavior, but it is ranked lower in importance than the previous options because it may not necessarily address the current situation. Moreover, even if Lara has not met up with patients outside of work before, it does not necessarily mean that her behavior is appropriate in this case.
5. Option C: Suggest to Lara that she speaks to her other colleagues to see what they think While it is important to seek advice and support from colleagues in difficult situations, this option is ranked lower in importance because it does not address the immediate concern of maintaining appropriate professional boundaries with the patient. Additionally, asking other colleagues for their opinion may not necessarily lead to a resolution of the situation, and may further complicate matters.

49

1. Option B: Discuss the comment made by the registrar/specialty trainee with your clinical supervisor* (Most important)

It is essential to seek guidance and support from a more senior colleague to help you understand the feedback and to identify if there is any truth to it. Your clinical supervisor can provide you with constructive feedback and help you develop an action plan to address any areas of concern.

1. Option C: Arrange to speak to the registrar/specialty trainee to discuss her feedback in more detail

It is important to have an open and honest conversation with the registrar/specialty trainee to understand their feedback better and to clarify any misunderstandings. This discussion can help you identify areas for improvement and develop strategies to enhance your performance.

1. Option A: Ask some of the other doctors working in the Emergency Department if they think you are slow

It may be helpful to seek feedback from your colleagues to gain a broader perspective. However, it is important to note that their opinions may not be representative or objective. Therefore, it is not the most reliable source of feedback.

1. Option D: Try to increase the number of patients you see in a shift

Increasing the number of patients you see in a shift may not necessarily address the root cause of the feedback you received. It is crucial to focus on the quality of care you provide and not just the quantity. Trying to rush through your tasks can lead to errors and may compromise patient safety.

1. Option E: Compare the number of patients you saw on a shift to the other FY1 doctors working in the department (Least important)

Comparing the number of patients you saw on a shift to other doctors can be misleading as each doctor's workload and patient case complexity can differ. It is essential to focus on your performance and seek feedback from those who work closely with you.

**50**

1. Option C: “I don't think that the ward experience alone will give you all of the clinical knowledge you will need.”

This is the most important consideration because it highlights the importance of attending the teaching sessions. As a FY1 doctor, it is essential to have a broad base of knowledge, which is not limited to the ward experience. Clinical teaching sessions are designed to provide additional learning opportunities and expose trainees to new topics, which will ultimately improve patient care. It is important to encourage the FY1 colleague to attend the sessions as they will be beneficial to their development as a doctor.

1. Option D: “Have you thought about discussing this issue with your clinical supervisor*?”

It is crucial to encourage the FY1 colleague to discuss their attendance at the teaching sessions with their clinical supervisor. It is possible that their workload is too high, and there are specific reasons why they are unable to attend. By discussing the issue with the clinical supervisor, the FY1 colleague may be able to find a solution that works for both their workload and attendance at the teaching sessions.

1. Option E: “Would it be helpful for me to explain what I find useful about the teaching sessions?"

It is important to offer support and guidance to the FY1 colleague, and explaining what has been helpful about the teaching sessions could encourage them to attend. However, this option is ranked lower as it is not as crucial as encouraging attendance or discussing the issue with the clinical supervisor.

1. Option B: “Both the teaching sessions and ward experience will be useful to develop your knowledge.”

While this option acknowledges that the ward experience is valuable, it is still important to attend the teaching sessions. Therefore, this option is ranked lower as it does not emphasize the importance of attending the teaching sessions as much as option C.

1. Option A: “It is up to you to decide how you prioritize your time.”

This option is ranked the least important as it does not provide specific guidance on how to manage the situation. As a FY1 doctor, it is important to encourage attendance at the teaching sessions and ensure that the colleague is aware of their importance. While it is ultimately up to the colleague to decide how to prioritize their time, it is important to provide guidance and support to ensure that they are developing their clinical knowledge as much as possible.

51

1. Option A: Thank Roisin for the invitation but explain that it would not be appropriate for you to attend This option is the most important consideration in this situation because it is important to maintain professional boundaries with patients. Attending a patient's social event could blur the lines between a professional and personal relationship and could potentially compromise the quality of care that the doctor provides. It is important to thank Roisin for the invitation, but also to explain politely why it would be inappropriate for the doctor to attend.
2. Option C: Politely decline Roisin's invitation, saying that you cannot attend This option is also important because it reiterates the importance of maintaining professional boundaries with patients. It is important to decline Roisin's invitation politely and clearly state the reasons why it would not be appropriate for the doctor to attend. This can help to prevent any further misunderstandings or awkwardness.
3. Option B: Invite other individuals from the medical team that has looked after Roisin to attend the party with you While this option could be a good compromise, it is not the most important consideration in this situation. Inviting other members of the medical team could help to maintain professional boundaries and prevent any potential misunderstandings, but it is still not appropriate for the doctor to attend the social event.
4. Option E: Explain that you are unable to attend, but buy Roisin a small gift While it is a kind gesture to buy Roisin a gift, it is not the most important consideration in this situation. Giving a gift could potentially be seen as an attempt to buy the patient's favor or to blur the lines between a professional and personal relationship.
5. Option D: Ask another doctor on the ward to manage Roisin's care until she is discharged This option is the least important consideration in this situation. It is not necessary to ask another doctor to manage Roisin's care just because the doctor cannot attend a social event. The doctor can continue to provide the same level of care as before and still maintain professional boundaries.

52

1. Option B: “Unfortunately, we have to prioritise patients based on their condition, which is why some patients have to wait longer than others.”

This option is the most important because it explains the reason why some patients have to wait longer than others, and it helps the patient understand the process. It also helps to manage the patient's expectations and ensures that they are aware of the reason for the delay.

1. Option E: “I understand it can be frustrating to wait, but rest assured the team are working hard to see people as quickly as they can."

This option acknowledges the patient's frustration and helps to empathize with the patient. It reassures the patient that the team is doing their best to see patients as quickly as possible.

1. Option A: “Please continue to wait patiently as the team are working as quickly as they can.”

This option asks the patient to be patient and wait for their turn to be seen. While it is important to let the patient know that the team is working hard, this option does not address the reason for the delay and may not be sufficient in managing the patient's frustration.

1. Option C: “You do not require urgent treatment, so please continue to wait.”

This option is not the most appropriate because it does not acknowledge the patient's frustration and may exacerbate their feelings of being unimportant. It is important to acknowledge the patient's concerns and explain the reason for the delay.

1. Option D: “It is unreasonable for you to shout in the waiting room. Unfortunately, you have to wait to be seen.”

This option is the least important because it is confrontational and may escalate the situation. It is important to address the patient's frustration in a calm and empathetic manner, rather than confronting the patient.

53

Ranking:

1. Option E: Establish the urgency of inserting the cannula before deciding what to do
2. Option A: Wait for the registrar/specialty trainee to finish in theatre so he can show you what to do
3. Option B: Watch an instructional video on how to cannulate children, before inserting the cannula
4. Option C: Phone the registrar/specialty trainee whilst he is in theatre to explain that you cannot insert the cannula
5. Option D: Try to cannulate the patient as instructed by the registrar/specialty trainee

Rationale: Option E is the most important consideration in this situation. As a doctor, it is crucial to determine the urgency of the situation before deciding on a course of action. If the cannulation is urgent, delaying it could potentially harm the patient. On the other hand, if it is not urgent, waiting for the registrar/specialty trainee to finish in theatre or watching an instructional video could be a better approach.

Option A is the second most important consideration as it is always better to have proper training and guidance before performing any medical procedure. Waiting for the registrar/specialty trainee to finish in theatre would ensure that the procedure is performed correctly and safely.

Option B is also important as it can provide some basic knowledge on how to cannulate children, which can be useful if the situation is not urgent.

Option C is not an ideal option, as it would delay the procedure and potentially harm the patient if the situation is urgent. However, if the situation is not urgent, it might be helpful to explain to the registrar/specialty trainee why you cannot insert the cannula and seek their guidance.

Option D is the least important consideration as it involves trying to perform the procedure without proper training, which can be dangerous and potentially harm the patient. It should only be considered if the situation is urgent and there are no other options available.

54

1. Option B: The impact of the radiologist’s behaviour on patient care The most important consideration in this situation should be patient care. The CT scan request may be essential to the patient's management, and any delay or refusal by the radiologist due to unprofessional behavior can have an adverse impact on the patient's health. Therefore, it is crucial to ensure that patient care is not compromised.
2. Option D: The radiologist’s past behaviour with other members of staff The radiologist's past behavior with other staff members is also significant because it indicates that the current incident is not an isolated one. The radiologist's rude behavior may be impacting the work environment, staff morale, and ultimately patient care. Therefore, it is important to take action to address this behavior.
3. Option A: That your FY1 colleague has requested not to mention it to anyone While it is important to respect your colleague's wishes, patient care and the working environment should take priority. It may be helpful to explain to your colleague that reporting this behavior can improve the work environment and ultimately benefit patient care. However, if your colleague insists on keeping the incident private, it may be necessary to respect their wishes.
4. Option C: That the radiologist is more senior than your FY1 colleague The radiologist's seniority should not be a significant consideration in this situation. While the radiologist may be more experienced, their behavior towards colleagues should always be professional and respectful. All staff members, regardless of their seniority, have a responsibility to maintain professional conduct in the workplace.
5. Option E: That you were not directly involved in the incident While you may not have been directly involved in the incident, you have a responsibility to address unprofessional behavior that impacts patient care and the work environment. As a healthcare professional, it is important to advocate for patients and colleagues and promote a safe and respectful work environment.

55

Ranking:

1. Option C: “I will attend to non-urgent tasks, but sick patients always take first priority.”
2. Option D: “If I don’t answer a bleep, please assume that I am with a sick patient and leave some time before trying again.”
3. Option A: “Please don’t bleep me again about non-urgent matters.”
4. Option E: “By constantly bleeping me, you are delaying me from helping you.”
5. Option B: “If you bleep again, I will ignore it and not complete the tasks.”

Rationale:

1. Option C: It is important to prioritize the care of sick patients as their condition can deteriorate rapidly. However, non-urgent tasks should not be neglected, and the FY1 should attend to them when they have completed their urgent tasks. This option acknowledges the nurse's request for help while still emphasizing the importance of patient care.
2. Option D: If the FY1 is with a sick patient, they may not be able to respond immediately to a bleep. It is important to communicate this to the nurse and suggest waiting for some time before trying again.
3. Option A: It is important to communicate with the nurse and explain that non-urgent tasks will be attended to, but the FY1 is currently prioritizing the care of a sick patient.
4. Option E: This option may be perceived as confrontational and may strain the professional relationship between the nurse and the FY1. It is important to remain professional and communicate effectively to resolve the situation.
5. Option B: Threatening to ignore bleeps from the nurse may cause further tension and delay the completion of tasks. It is important to maintain professionalism and work collaboratively with the nursing staff to provide the best care for patients.

56

Ranking:

1. Option B: Report to the Foundation Programme Director the lack of response from Mr Jones
2. Option C: Contact Mr Jones' secretary, in order to find Mr Jones and discuss your induction meeting
3. Option D: Ask the Foundation Programme Director to assign you a new educational supervisor
4. Option E: Ask the hospital switchboard to transfer your call to Mr Jones' mobile telephone number
5. Option A: Refrain from attempting to make contact with Mr Jones, waiting for him to contact you

Rationale:

Option B is the most important consideration as it involves informing the appropriate authority, the Foundation Programme Director, about the lack of response from your assigned supervisor. The Foundation Programme Director is responsible for ensuring that all trainees receive adequate support and supervision, and they need to be made aware of the situation to take appropriate action. This will also help to document the lack of response from Mr Jones, which could be useful in the future.

Option C is the next important consideration, as it involves actively trying to locate Mr Jones and discuss the induction meeting with him. This shows initiative on your part and demonstrates that you are actively seeking solutions to the problem.

Option D is also an important consideration, as it involves requesting a new educational supervisor if the current one is not responding. However, this should only be considered after options B and C have been exhausted.

Option E is a less important consideration, as it involves trying to contact Mr Jones directly. This may not be appropriate as he may not be available or may not want to be contacted outside of work hours. It is also better to follow official channels of communication.

Option A is the least important consideration, as it involves waiting for Mr Jones to contact you. This may not be a practical solution as you need support and supervision in your role as an FY1 doctor.

57

Ranking:

1. Option B: Return to the ward immediately to take the blood sample
2. Option D: Contact the doctor covering the ward, to explain the situation
3. Option A: Take the blood sample when you return to work tomorrow morning
4. Option C: Send a text message to the doctor who is currently covering the ward
5. Option E: Phone the ward directly and leave a message with the nursing team

Rationale:

Option B is the most important consideration in this situation as the patient requires an urgent blood sample which needs to be taken today. It is important to ensure that the patient receives the necessary care and treatment in a timely manner. Therefore, returning to the ward immediately to take the blood sample is the best course of action.

Option D is the second most important consideration as it is important to communicate the situation to the doctor covering the ward to ensure continuity of care for the patient. The doctor covering the ward can take appropriate action and ensure that the patient receives the necessary care and treatment.

Option A is the third most important consideration as it may delay the necessary care and treatment for the patient. However, if returning to the ward immediately is not feasible due to safety concerns or other reasons, taking the blood sample when returning to work the next day may be the next best option.

Option C is the fourth most important consideration as text messages may not always be received promptly or may be missed altogether. Therefore, it is not the most reliable method of communication in urgent situations.

Option E is the least important consideration as leaving a message with the nursing team may not be the most effective method of communication in this situation. The nursing team may not have the necessary information or authority to take appropriate action for the patient.

58

1. Option C: Whether there are other doctors who could stay for another three hours The most important consideration is whether there are other doctors who could stay for another three hours. Patient safety must be the top priority, and it is important to ensure adequate staffing levels to maintain safe patient care. If there are other doctors available who can stay for the three hours, then it may be appropriate to delegate the responsibility to them. However, if there are no other doctors available, then it may be necessary for the FY1 doctor to stay for the extra shift.
2. Option A: The length of the shift you have just worked The length of the shift worked is an important consideration as it can affect the FY1 doctor's ability to function effectively and safely. Fatigue can impair decision-making and increase the risk of medical errors. If the FY1 doctor has already worked a long shift, then they may not be able to safely provide good patient care during the extra three hours. Therefore, the length of the shift worked should be considered when deciding whether to stay for the extra shift.
3. Option B: The frequency of you being asked to work extra hours The frequency of being asked to work extra hours is an important consideration as it can affect job satisfaction and work-life balance. However, patient safety must always be the top priority, and sometimes, it may be necessary to work extra hours to ensure safe patient care. Therefore, the frequency of being asked to work extra hours should be considered, but it should not be the primary factor in the decision-making process.
4. Option D: How much you will get paid for the extra work The amount of pay for the extra work is important to consider, but it should not be the primary factor in the decision-making process. Patient safety must always be the top priority, and the FY1 doctor should not prioritize their pay over the safety and well-being of their patients.
5. Option E: Your social life commitments for the evening Social life commitments are the least important consideration in this situation. While it is understandable that the FY1 doctor may have plans for the evening, patient safety must always be the top priority, and social commitments should not be prioritized over patient care. The FY1 doctor should consider rescheduling their plans for another time or delegate responsibilities to another doctor if possible.

59

Ranking:

1. Option C: “Sorry, I know that this was my mistake, but I think we should focus on the correct patient having a renal review.”
2. Option E: “Please can you review the correct patient, it should only take a few minutes.”
3. Option B: “Sorry I know that was my fault, but do you have any time to review the correct patient now?”
4. Option A: “That was a silly mistake. We all make mistakes though, don't we?”
5. Option D: “I thought you would double check before spending an hour reviewing the patient."

Rationale: Option C is the most important because it shows ownership of the mistake and places the focus on correcting the error and ensuring the correct patient receives appropriate care. It also acknowledges the registrar's time and effort, which may help to defuse any frustration or annoyance they are feeling.

Option E is the second most important because it provides a solution to the problem by requesting that the registrar review the correct patient. It also acknowledges that the review should only take a few minutes, which may help to alleviate any concerns the registrar has about being further delayed.

Option B is the third most important because it acknowledges the mistake and asks for the registrar's assistance in reviewing the correct patient. However, it does not address the issue of the registrar's time or effort, which may leave them feeling annoyed or frustrated.

Option A is the fourth most important because it minimizes the mistake by referring to it as a "silly mistake" and does not acknowledge the registrar's time or effort. This response may further irritate the registrar.

Option D is the least important because it is confrontational and may escalate the situation. It also places the blame on the registrar for not double-checking, which is not a constructive or helpful approach to resolving the issue.

60

Ranking of options:

1. Option C: Contact the consultant immediately to explain the situation
2. Option B: Explain to Simon that he should not conduct the review if he is under the influence of alcohol
3. Option A: Suggest to the nurse that she asks Simon whether he has been drinking
4. Option D: Ask a specialty trainee from another ward to review the patient with you
5. Option E: Continue with the review, but explain to Simon afterwards that he smells of alcohol

Rationale: Option C is the most important consideration in this situation as it involves contacting the consultant who is responsible for the patient's care. The consultant can make a decision about the management of the situation and ensure that the patient receives appropriate care. The consultant may also need to make arrangements for a replacement doctor to conduct the review.

Option B is the second most important consideration as it involves addressing the issue with Simon directly. It is important to explain to Simon that he should not conduct the review if he is under the influence of alcohol, as this could compromise patient safety and put the patient at risk. Simon may need support and assistance, and this can be provided through occupational health services.

Option A is the third most important consideration as it involves gathering more information about the situation. By suggesting that the nurse asks Simon whether he has been drinking, it may be possible to confirm the nurse's suspicion and provide more information to the consultant. However, this option may not be appropriate if the situation is urgent and requires immediate action.

Option D is the fourth most important consideration as it involves seeking assistance from another doctor. This may be necessary if the situation is urgent and requires immediate action. However, it is important to ensure that the doctor who is asked to assist is competent and has the necessary skills to manage the situation.

Option E is the least important consideration as it involves continuing with the review despite the suspicion that Simon has been drinking. This option is not appropriate as it could compromise patient safety and put the patient at risk. It is important to address the issue with Simon directly and seek assistance from the consultant if necessary.

61

1. Option A: Ask the patient and his relatives if you can discuss his progress when you are back from the teaching session (1= Most important)

This option prioritizes patient care and communication by acknowledging the patient and their relatives' request for an update on the patient's progress. It also recognizes the importance of attending the teaching session to maintain competence. By offering to discuss the patient's progress after the teaching session, the doctor demonstrates a commitment to patient care while also fulfilling their educational requirements.

1. Option B: Ask one of your colleagues if they would be willing to have a discussion with the patient and his relatives (2)

If the doctor cannot personally attend to the patient and their relatives, asking a colleague to do so is a reasonable alternative. This option maintains the continuity of care and ensures that the patient's concerns are addressed in a timely manner. However, it may not be possible to find a colleague who is available to help, or the patient and their relatives may prefer to speak with the doctor directly.

1. Option E: Inform the nurse that you are busy and continue on your way to the teaching session (3)

While it is important to attend the teaching session, ignoring the patient and their relatives' request for an update is not appropriate. This option risks creating a negative impression of the doctor and the hospital's care for the patient. However, if the doctor genuinely cannot take the time to speak with the patient and their relatives, it is better to be honest with the nurse about their commitments.

1. Option D: Discuss the patient’s progress with him and his relatives and attend the teaching session late (4)

This option risks being late to the teaching session, which may have consequences for the doctor's competence progression. While it is important to attend the session, it is also essential to maintain a professional attitude and to be respectful of colleagues' time. Additionally, the doctor may not have enough time to address the patient and their relatives' concerns fully.

1. Option C: Miss the teaching session so that you can discuss the patient’s progress with him and his relatives (5= Least important)

This option ignores the importance of continuing professional development and competence progression. While patient care is crucial, missing a teaching session entirely to discuss a patient's progress is not an appropriate solution. The doctor's competence progression may suffer, and the hospital may be short-staffed for the session.

62

Ranking:

1. Option C: Ask the consultant if there are any strategies that you could use to manage the patient's abusive behaviour
2. Option B: Ask your FY1 colleague if she is available to help you examine the patient
3. Option A: Request that the consultant attends the examination of the patient with you
4. Option E: Explain to the consultant why you found the interaction with the patient last week extremely challenging
5. Option D: Inform the consultant that you do not want to conduct the examination following your last experience with the patient

Rationale:

1. Option C: It is important to ask the consultant for strategies to manage the patient's abusive behavior. Patients with advanced dementia may exhibit challenging behaviors, and it is important to know how to deal with these situations. By seeking guidance from the consultant, the FY1 doctor can learn how to manage similar situations in the future, and provide better care to the patient.
2. Option B: If the FY1 doctor feels uncomfortable conducting the examination alone, it is appropriate to ask a colleague for help. The colleague can provide support during the examination and help manage any challenging behavior from the patient.
3. Option A: If the FY1 doctor still feels uncomfortable conducting the examination alone or with a colleague, it is appropriate to request the consultant to attend the examination. However, this should only be considered as a last resort, as it is important for the FY1 doctor to learn how to manage difficult situations and gain confidence in their clinical skills.
4. Option E: It is important to communicate to the consultant why the previous interaction with the patient was challenging, as this may help the consultant understand the FY1 doctor's perspective. However, this option is less important than seeking guidance and support from the consultant or a colleague, as it does not directly address how to manage the patient's behavior.
5. Option D: It is not appropriate for the FY1 doctor to refuse to conduct the examination without seeking guidance or support first. Refusing to conduct the examination could compromise patient care, and it is important for the FY1 doctor to learn how to manage difficult situations and gain confidence in their clinical skills.

63

1. Option E: “You should respect the patient’s decision regardless of your beliefs.” Respecting the patient's decision is the most important consideration in this scenario. As a healthcare professional, it is our duty to provide non-judgmental and unbiased care to all patients, irrespective of our personal beliefs. The patient has the right to make decisions about their own body, and we should respect and support their choices.
2. Option A: “You shouldn’t let your feelings about termination be known to the patient.” It is important not to let personal feelings about termination influence the care provided to the patient. Patients trust healthcare professionals to provide them with accurate information and support, and any bias or negative attitude towards their decisions may affect the quality of care provided.
3. Option C: “It is unprofessional to be judgemental towards others who have different beliefs to you.” Being judgmental towards patients with different beliefs is unprofessional and may damage the therapeutic relationship between the healthcare professional and the patient. As healthcare professionals, we must maintain professionalism at all times and provide care that is respectful and non-discriminatory.
4. Option B: “You’re entitled to your own opinion.” While healthcare professionals are entitled to their own opinions, it is important to not let personal beliefs and opinions affect patient care. It is crucial to maintain a professional and unbiased approach towards patient care.
5. Option D: “Some terminations are not due to the choice of the patient, it could be medically indicated.” While it is true that some terminations may be medically indicated, this consideration is less relevant in this scenario as the reason for the termination is not specified. Additionally, regardless of the reason for the termination, it is important to respect the patient's decision and provide non-judgmental care.

64

Ranking:

1. Option E: Ask Dr Grey for any further background information that might help you understand Adeel’s presentation
2. Option B: Inform Dr Grey that you may need to ask him some questions after Adeel’s review to check your understanding
3. Option D: Ask Dr Grey to supervise the review as Adeel is not your patient
4. Option C: Suggest to Dr Grey that you will complete a very basic review of Adeel
5. Option A: Explain to Dr Grey that Adeel is not your patient so you do not feel comfortable conducting the review

Rationale:

1. Option E: Asking for further background information will help to ensure that you have a better understanding of the patient's history, which will aid in the accuracy of the review. This information will also help in identifying any potential red flags that need to be addressed.
2. Option B: Informing Dr Grey that you may need to ask him some questions after Adeel’s review will ensure that you have the opportunity to clarify any doubts you have and seek further guidance if needed. This approach will help you in identifying any gaps in your knowledge and improve your learning.
3. Option D: Asking Dr Grey to supervise the review will provide an opportunity to have someone with more experience overseeing the review, and this will help mitigate any risks that might result from a lack of familiarity with the patient's history.
4. Option C: Suggesting to Dr Grey that you will complete a very basic review is a good option when time is of the essence, but it is important to ensure that all necessary information is obtained before taking this approach.
5. Option A: Explaining to Dr Grey that you are not comfortable conducting the review is the least important option. It is essential to make sure that you are competent in the tasks you are performing, but this should not come at the cost of patient care.

65

1. Option B: That Samira could pass on the illness to patients or colleagues - This is the most important consideration in this situation as patient safety is the highest priority. Samira could be contagious and could spread the illness to vulnerable patients or other colleagues, which could potentially have serious consequences. She needs to be advised to leave work immediately and seek medical attention, especially if she has symptoms such as fever, vomiting, or diarrhea.
2. Option C: That Samira should be resting in order to recover from the illness - Samira's health and well-being are also important considerations, and she should not be working if she is unwell. She needs rest and time to recover from the illness.
3. Option D: Whether there are enough staff to see the patients should Samira be off sick - While it is important to ensure that patient care is not compromised, it should not take precedence over patient safety or Samira's health. If Samira is unwell and potentially contagious, patient care may be adversely affected by her being present. Therefore, the decision to stay at home and rest should be prioritized over staff shortages.
4. Option A: The ability of Samira to meet her educational needs - Although education is important, it should not be prioritized over patient safety, Samira's health, or the needs of the hospital. The teaching session can be caught up at a later time, and it is not worth risking patient safety or worsening Samira's health.
5. Option E: That Samira has already missed several teaching sessions this year - While it is important to attend teaching sessions, it should not be prioritized over patient safety, Samira's health, or the needs of the hospital. Missing a teaching session is not worth risking patient safety or worsening Samira's health. The missed teaching sessions can be caught up at a later time.

66

1. Option B: Explain that this task has taken you longer than 30 minutes previously, and you need to leave at 17:00 for a meeting. This option is the most important because it is crucial to communicate your situation to the registrar/specialty trainee. By explaining that the task has taken longer previously and you need to leave at 17:00 for a meeting, you are being transparent and setting clear expectations. This helps to avoid any misunderstanding or miscommunication that could lead to potential problems down the line.
2. Option D: Ask if you can help to find a different colleague to help with the task as you need to leave at 17:00 for a meeting. If the registrar/specialty trainee is insistent that the task needs to be done today, then asking for help to find a different colleague is the next best option. This shows that you are willing to help, but you also need to prioritize attending the meeting with your educational supervisor. It is important to make sure that the task is completed, but it does not necessarily have to be completed by you at this particular moment.
3. Option C: Agree to complete the task. This option is acceptable, but only if you are confident that you can complete the task in the time remaining before your meeting. However, if you feel rushed or stressed, this may not be the best option as it could compromise the quality of your work or even cause you to be late for your meeting.
4. Option E: Ask the registrar/specialty trainee if she can find another colleague to complete the task. This option is less important because it puts the responsibility back on the registrar/specialty trainee to find another colleague to complete the task. As a FY1 doctor, it is important to be proactive and take responsibility for tasks assigned to you, rather than passing them off to others.
5. Option A: Inform the registrar/specialty trainee that you are unable to help. This option is the least important because it does not offer any solution or alternative. It is important to be a team player and help out whenever possible, as long as it does not compromise patient safety or interfere with your other responsibilities. By simply informing the registrar/specialty trainee that you are unable to help, you are not contributing to finding a solution to the problem at hand.

67

1. Option A: You are responsible for completing a thorough handover at the end of your shift Completing a thorough handover is the most important consideration in this situation. It is essential to ensure that patient care is not compromised and that Lucas has all the necessary information to continue caring for the patients. Providing a comprehensive handover ensures that Lucas can make informed decisions about the care of the patients and prioritize their needs accordingly. It is a critical responsibility of an FY1 doctor to hand over care effectively and safely to ensure continuity of care and patient safety.
2. Option D: The level of detail required to effectively explain the shift handover information It is important to provide adequate detail during the handover to ensure that Lucas understands the patients' conditions, their management plans, and any potential issues that could arise. This includes any significant events that occurred during the shift, changes in patient conditions, and any interventions that were performed. However, this consideration is of secondary importance to Option A, as the thoroughness of the handover is a more significant factor in ensuring patient safety.
3. Option C: If there is another staff member available who could share the information with Lucas when he arrives If there is another staff member available who could share information with Lucas, this would be beneficial, but it is not essential. The priority is for the handover to occur as soon as possible to ensure continuity of care. However, if another staff member is available to assist, it may help to alleviate any concerns about the quality of the handover due to time constraints.
4. Option B: The inconvenience to your family if you are late While it is important to maintain a work-life balance and keep personal commitments, in this situation, the priority is to provide a comprehensive handover to ensure patient safety. Any inconvenience to the doctor's family is a secondary consideration and should not affect the handover's quality or timing.
5. Option E: It is not your responsibility to ensure that Lucas arrives on time for his shift While it is not the doctor's responsibility to ensure that Lucas arrives on time for his shift, this consideration is of the least importance. The primary responsibility is to complete the handover thoroughly to ensure patient safety, and any issues with Lucas's arrival time can be addressed separately.

68

1. Option B: Escalate the relatives' request to the on-call consultant - This is the most important consideration as the situation requires a decision to be made by a senior doctor with more experience and authority to handle sensitive issues. The consultant can also provide guidance on how to approach the situation, respecting the cultural and religious beliefs of the family while ensuring that appropriate procedures are followed.
2. Option A: Telephone Claire to ask that she comes back to the hospital to sign the patient's death certificate - This is important because Claire, who was treating the patient earlier, should ideally sign the death certificate as she has knowledge of the patient's medical history and can ensure the accuracy of the information. However, it is not as urgent as Option B as the on-call consultant needs to be informed first.
3. Option E: Contact the ward manager to see if there are any other doctors in the hospital who are able to sign the death certificate - This is also important because it is necessary to find another doctor who is authorized and able to sign the death certificate if neither Claire nor the on-call consultant are available. The ward manager may be able to provide this information quickly and help to avoid any unnecessary delays.
4. Option D: Sign the patient's death certificate - This is not ideal as the doctor signing the certificate should have knowledge of the patient's medical history and clinical condition at the time of death. Therefore, if Claire or another doctor who knows the patient is not available, it is better to escalate the situation to a senior doctor before signing the certificate.
5. Option C: Tell the relatives that Claire has left the hospital and will not be able to return to sign the death certificate until her next shift on Monday - This is the least important option as it is not an appropriate response to the situation. It does not address the family's concerns and may cause unnecessary distress, especially considering their cultural and religious beliefs.

69

1. Option B: Ask your FY2 colleague if she could allocate the ward tasks more equally between the two of you - This is the most important consideration as it directly addresses the issue. It is important to communicate with your colleague and express your concerns in a professional and non-confrontational manner. By doing so, you may be able to come up with a more equitable solution to task allocation that benefits both of you.
2. Option D: Talk to your clinical supervisor* about the situation - If after discussing the issue with your colleague, the situation does not improve, then it is appropriate to seek the advice and support of your clinical supervisor. They can provide guidance on how to handle the situation and may be able to intervene if necessary.
3. Option A: Monitor the situation for a bit longer to see if it improves - It is important to give your FY2 colleague the benefit of the doubt and observe the situation for a while. However, if you find that the task allocation remains uneven, it is important to take action.
4. Option E: Ask your FY2 colleague if there is a reason why she is allocating more of the ward tasks to you - This option is less important than Option B because it assumes that there is a valid reason for the unequal task allocation. While it is important to understand your colleague's perspective, it should not be used as an excuse for unfair treatment.
5. Option C: Ask another colleague on the ward if they think your FY2 colleague is being unfair with allocating the ward tasks - This option is the least important because it may create unnecessary conflict and does not directly address the issue with your colleague. It is better to address the issue directly with your colleague first before involving others.

70

1. Option B: “I’m completing an urgent discharge summary, I will change the prescription after.” This option is the most important because the urgent discharge summary takes priority as the patient's transport has already been booked. Delaying the discharge summary could cause unnecessary harm or discomfort to the patient, and it is crucial to complete it in a timely manner. It is also important to acknowledge the nurse's request for the prescription change and assure them that it will be addressed as soon as possible.
2. Option E: “I will come and find you after I have completed this task, to discuss your request.” This option is also important because it demonstrates a commitment to addressing the nurse's request while also prioritizing the urgent discharge summary. It is important to communicate clearly and follow up on the request as soon as possible to ensure that patient care is not compromised.
3. Option C: “I’m completing an urgent discharge summary, is anyone else able to help?” While it is important to seek help from colleagues when necessary, in this situation, it may not be the most efficient option. It is crucial to complete the discharge summary in a timely manner, and finding someone else to complete the task could cause unnecessary delays. However, if there is a colleague available who can help, it may be a good option to consider.
4. Option A: “I already have a lot of other requests to deal with.” While it is true that there may be other tasks that need to be completed, it is not a productive or effective response in this situation. It does not address the urgent nature of the discharge summary or the nurse's request for the prescription change. It is important to prioritize tasks and communicate effectively to ensure that patient care is not compromised.
5. Option D: “I’ll never get any tasks done if I keep getting interrupted.” This option is the least important because it does not address the urgent nature of the discharge summary or the nurse's request for the prescription change. While it is important to minimize interruptions, it is also important to prioritize tasks and communicate effectively to ensure that patient care is not compromised.

71

1. Option B: Whether the information provided in the patient file is understood The most important consideration is to ensure that the information provided in the patient file is understood. As the next healthcare provider responsible for this patient, it is essential to have a clear understanding of the patient's management plan, including any interventions or treatments that have been started. This will enable the FY1 to provide appropriate care for the patient and make informed decisions about their ongoing management.
2. Option D: Your need to start completing your tasks As a healthcare provider, there are multiple tasks and responsibilities that need to be completed during a night shift. It is important to prioritize tasks, especially those that directly affect patient care. In this scenario, after attempting to read the patient file, it is important to continue with other tasks that need immediate attention, while attempting to clarify any confusion about the patient management plan as soon as possible.
3. Option C: The reason why the FY1 had to rush the last section of the patient file While it is important to understand the reason why the previous FY1 had to rush the last section of the patient file, it should not be prioritized over understanding the patient's management plan and completing immediate tasks. However, if there is time, it may be helpful to discuss with the previous FY1 to understand why they rushed the last section and if there are any further important details that need to be communicated.
4. Option E: Other ways you could find out about patient management plan While there may be other ways to find out about the patient management plan, such as speaking with the nursing staff or reviewing the patient's electronic medical records, these options should only be considered after attempting to read the patient file. Additionally, these options may not provide a complete understanding of the patient's management plan, as the patient file should contain all necessary information.
5. Option A: How the FY1 will handle receiving the feedback about his handwriting While providing feedback to colleagues is an important aspect of professional development, it should not be prioritized over understanding the patient's management plan and completing immediate tasks. However, if there is time, it may be appropriate to provide feedback to the previous FY1 in a constructive and supportive manner, with the aim of helping them improve their record-keeping skills.

72

Ranking of importance in the management of this situation:

1. Option A: Inform Dr Hadi that the data was fabricated by Zara
2. Option E: Suggest to Zara that she admits to Dr Hadi that she fabricated the data
3. Option C: Tell Dr Hadi that there was a mistake with the data, so you and Zara will need to collect some more before presenting at the conference
4. Option D: Attend the conference with Zara, but refuse to present any of the findings
5. Option B: Tell Dr Hadi that you did not have any input into the audit so Zara should attend the conference alone

Rationale:

Option A is the most important consideration in this situation because it is crucial to be honest and transparent with Dr Hadi about the fabrication of data by Zara. Falsifying data is a serious breach of ethics and professional conduct, and it is the duty of the FY1 doctors to report such conduct. This is also important to protect the reputation of the hospital and the medical profession.

Option E is the second most important consideration as it involves taking action to rectify the situation by encouraging Zara to come forward and admit to Dr Hadi that she fabricated the data. This will demonstrate accountability and responsibility for the actions and help in restoring the trust of the clinical supervisor and the hospital.

Option C is the third most important consideration as it involves addressing the incomplete data collection by informing Dr Hadi about the mistake and suggesting that further data collection is necessary before presenting the findings. This is important to ensure that the conclusions drawn from the audit are based on accurate and reliable data.

Option D is the fourth most important consideration, as it involves attending the conference but refusing to present the fabricated findings. This option can help to avoid any potential damage to the hospital's reputation, but it does not address the issue of falsified data.

Option B is the least important consideration as it involves distancing oneself from the situation and placing the blame solely on Zara. This is not a proactive approach to addressing the issue of falsified data and does not reflect the professional values and ethics of the medical profession.

73

1. Option A: “I am sorry to disturb you, but I am worried about this patient” - This should be the most important consideration in this situation. The patient's well-being should always be the top priority, and it is crucial to convey your concern to the surgical registrar/specialty trainee. By expressing your concern, you can communicate the urgency of the situation and the need for timely intervention.
2. Option B: “Can you explain why you feel that I have made a wrong decision?” - This option could be helpful to understand the surgical registrar/specialty trainee's perspective and clarify any misunderstandings. However, it should not take precedence over Option A as it may delay the patient's treatment.
3. Option C: “Would you like me to explain why I bleeped you?” - This option could be useful to address any misunderstandings about the reason for the bleep. However, it is not as urgent as Option A, and it could be addressed later.
4. Option E: “My FY1 colleagues have said that you are very rude” - It is not professional to involve colleagues in a personal conflict, and it may worsen the situation. This option should be avoided as it does not contribute to the patient's care.
5. Option D: “I feel that you are being very rude” - This option should be avoided as it could escalate the conflict further, and it does not address the patient's care. The focus should be on resolving the situation and ensuring the patient receives timely and appropriate treatment.

74

1. Option D: That your FY1 colleague is breaching patient confidentiality - This is the most important consideration in this situation. Patient confidentiality is of utmost importance in healthcare, and any breach of this could lead to legal and ethical issues for both the doctor and the hospital. The colleague should be reminded of their responsibility to maintain patient confidentiality and the consequences of breaching it. Additionally, the breach should be reported to the appropriate authorities to ensure that it is dealt with appropriately.
2. Option B: That the relatives of the patient that is being spoken about may be in the canteen - The presence of the patient's relatives in the canteen adds another layer of complexity to the situation. They have a right to know that their loved one's privacy has been breached, and it is important to ensure that they are informed of what has happened and that appropriate steps are taken to prevent it from happening again.
3. Option E: That patients and relatives in the canteen may not know that your FY1 colleague is speaking about a patient - While it is true that some patients and relatives in the canteen may not know what is happening, it is still important to consider their right to privacy and confidentiality. They may not want to hear about the medical conditions of other patients, and it is important to ensure that their privacy is protected as well.
4. Option A: That you have already attempted to say something to your FY1 colleague, and she has dismissed you - While it is important to attempt to address the situation with the colleague, the fact that they have dismissed you does not mean that the issue should be dropped. Other options should be explored, such as speaking to a senior member of staff or reporting the breach of confidentiality to the appropriate authorities.
5. Option C: That there are lots of other members of medical staff that could make your FY1 colleague aware of the issue - While there may be other members of staff who could address the issue with the colleague, it is ultimately the responsibility of the person who has witnessed the breach of confidentiality to take action. This does not mean that other staff members should not be informed, but it should not be relied upon as the sole solution to the problem.

75

1. Option E: Ask a colleague for advice on how to proceed As a FY1 doctor, it is important to recognize one's limitations and seek advice from more experienced colleagues when encountering challenging situations. Seeking advice from a senior colleague or a more experienced healthcare professional is a crucial aspect of clinical practice, and it demonstrates a willingness to learn and improve patient care. In this scenario, seeking advice from a colleague could help in identifying new approaches or techniques that can help in inserting the cannula.
2. Option D: Ask another colleague to assist you If the patient is becoming agitated and losing patience, it is important to act quickly to reduce their distress. Asking another colleague to assist in inserting the cannula can help expedite the process and reduce the patient's anxiety. Additionally, working collaboratively with colleagues is essential in providing high-quality patient care and can provide opportunities for learning and development.
3. Option B: Leave it in the evening handover for a colleague to complete Handover is a crucial part of clinical practice, and it is important to communicate effectively with colleagues to ensure that patients receive appropriate care. If attempts to insert the cannula have been unsuccessful, it may be appropriate to leave it in the evening handover for a colleague to complete. However, this option should only be considered if there is no immediate need for antibiotics or if the patient's condition is stable.
4. Option A: Attempt to insert the cannula again Attempting to insert the cannula again may not be the best option if previous attempts have been unsuccessful, as it may cause further distress to the patient and delay appropriate treatment. Moreover, repeated attempts to insert the cannula may increase the risk of complications, such as infection or hematoma.
5. Option C: Call the registrar/specialty trainee again and ask him to reconsider his decision If a registrar/specialty trainee has refused to assist in inserting the cannula, it may not be productive to call again and ask them to reconsider their decision. It is essential to respect the hierarchy and responsibilities of different healthcare professionals, and if a senior colleague is not available to assist, it is better to seek help from another source. Additionally, it is important to understand that it is the responsibility of a junior doctor to insert a cannula, and if there is difficulty, it is appropriate to seek advice or assistance from colleagues.
